# Childhood Asthma and Allergy Are Related to Accelerated Epigenetic Aging

**DOI:** 10.1111/all.16583

**Published:** 2025-05-10

**Authors:** Miriam Leskien, Elisabeth Thiering, Zhebin Yu, Anke Huels, Yueli Yao, Simon Kebede Merid, Olena Gruzieva, Stephan Weidinger, Melanie Waldenberger, Annette Peters, Erik Melén, Marie Standl

**Affiliations:** ^1^ Institute of Epidemiology Helmholtz Zentrum München ‐ German Research Center for Environmental Health Neuherberg Germany; ^2^ Medical Faculty, Institute for Medical Information Processing, Biometry, and Epidemiology Ludwig‐Maximilians‐Universität München Munich Germany; ^3^ German Center for Child and Adolescent Health (DZKJ) Munich Germany; ^4^ Division of Metabolic and Nutritional Medicine, Dr. von Hauner Children's Hospital University of Munich Medical Center Munich Germany; ^5^ Institute of Environmental Medicine Karolinska Institutet Stockholm Sweden; ^6^ Department of Epidemiology, Rollins School of Public Health Emory University Atlanta Georgia USA; ^7^ Gangarosa Department of Environmental Health, Rollins School of Public Health Emory University Atlanta Georgia USA; ^8^ Department of Clinical Sciences and Education, Södersjukhuset Karolinska Institutet Stockholm Sweden; ^9^ Sachs' Children and Youth Hospital Södersjukhuset Stockholm Sweden; ^10^ Centre for Occupational and Environmental Medicine Stockholm Sweden; ^11^ Department of Dermatology, Venereology, and Allergology University Hospital Schleswig‐Holstein Kiel Germany; ^12^ Research Unit Molecular Epidemiology Helmholtz Zentrum München ‐ German Research Center for Environmental Health Neuherberg Germany; ^13^ Chair of Epidemiology Ludwig‐Maximilians University Munich Germany; ^14^ German Center for Lung Research (DZL) Munich Germany

**Keywords:** allergic diseases, epidemiology, epigenetic aging, epigenetic clock, pediatrics

## Abstract

**Background:**

Few studies showed associations of childhood allergic diseases with epigenetic aging using traditional clocks trained mainly on adults. Tracking DNA methylation variation early in life has suggested poor performance of these clocks in children. Therefore, we aim to elucidate the association between allergic diseases and epigenetic age using a pediatric clock.

**Methods:**

We used data from the German LISA birth cohort study at six (*N* = 234) and ten (*N* = 227) years. DNA methylation was measured in blood using the Infinium Methylation EPIC BeadChip. We calculated epigenetic age using the pediatric clock developed by Wu et al. (Aging 2019) (median absolute error = 0.04 years, Spearman correlation with chronological age *r* = 0.75). Linear mixed models were used to examine longitudinal associations of epigenetic age acceleration with doctor‐diagnosed asthma, rhinitis, and eczema, or a combination thereof (“any allergy”) as well as aeroallergen sensitization. Replication was performed in BAMSE at the 8‐year follow‐up (*N* = 625) using linear models. Additionally, epigenetic age in adults from KORA F4 was estimated using Horvath's clocks and associations with allergic diseases were tested applying linear models.

**Results:**

Having any allergy was significantly associated with a mean epigenetic age acceleration of 0.34 years (95% CI = [0.06; 0.63]) using Wu's clock in LISA. Associations with consistent effect directions were found for allergic rhinitis, asthma, and eczema. No associations with aeroallergen sensitization were observed. In BAMSE, an inverse association of epigenetic age acceleration with eczema was found (−0.52 years, 95% CI = [−0.97; −0.07]). In KORA, hay fever was significantly associated with accelerated epigenetic age when using the Horvath pan‐tissue clock (1.05 years, 95% CI = [0.21; 1.89]).

**Conclusions:**

We found an increase in epigenetic age in children with allergic diseases from LISA. Our results suggest that epigenetic age acceleration seems to be related to the persistent burden of allergic diseases, but not to non‐symptomatic aeroallergen sensitization.

## Graphical abstract

We used a pediatric epigenetic clock to assess epigenetic age in children from a German birth cohort. Accelerated epigenetic aging was found to be related to the presence of allergic diseases like asthma, rhinitis, or eczema, but not to non‐symptomatic aeroallergen sensitization. This was supported by an epigenetic age acceleration gradient in response to allergic disease persistence and allergic multimorbidity. BAMSE, Swedish abbreviation for Children, Allergy, Milieu, Stockholm, Epidemiology; KORA F4, German population‐based Cooperative Health Research in the Region of Augsburg; LISA, Life‐style factors on the Development of the Immune System and Allergies in East and West Germany.

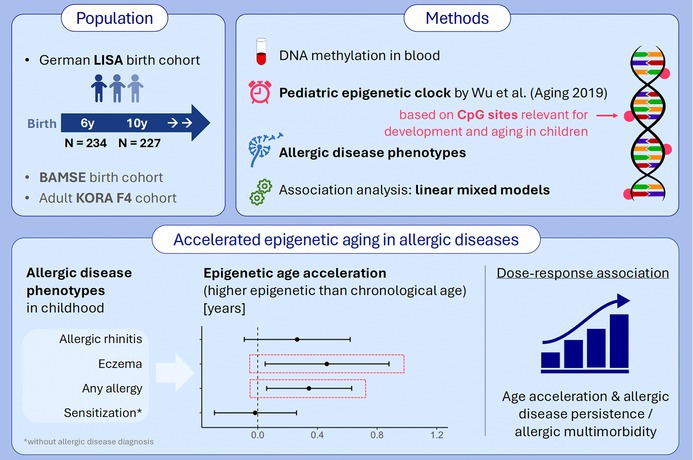

## 1 Introduction

The rise in the prevalence of allergies over the last decades [1] underlines the important role of environmental and lifestyle factors in the pathogenesis [2]. DNA methylation (DNAm) is suggested to be an important mechanism linking environmental exposures, genetics, and allergic disease risk. Epigenome‐wide association studies identified differentially methylated 5'‐Cytosine‐phosphate‐Guanine‐3′ (CpG) sites to be associated with atopy [3, 4, 5], childhood asthma [6], or any allergic disease with sensitization [7].

In contrast to investigating the role of specific CpG sites, epigenetic clocks reflect age‐dependent changes of the methylome. This method is based on the DNAm levels of CpG sites that were selected to predict chronological age and are relevant for development and aging. It estimates epigenetic age, hereinafter referred to as “DNAmAge.” Epigenetic aging can be influenced by lifestyle and environmental factors [8], and epigenetic aging clocks have been linked to age‐related health outcomes [9]. When biological age is higher than chronological age, this is referred to as epigenetic age acceleration (EAA). Interestingly, EAA is suggested to play a role not only in age‐related diseases but also in diseases commonly emerging in childhood, like allergy and asthma [10, 11].

To date, only a few studies investigated associations of EAA with allergic diseases in childhood [10, 11, 12], using epigenetic clocks mainly trained on adults. One study reported cross‐sectional associations of EAA with higher total serum immunoglobulin E (IgE), higher odds of atopic sensitization, and asthma at mid‐childhood [10]. Another study observed an increased epigenetic age of the nasal methylome in children with asthma [11], and recently, accelerated epigenetic age was also found in children with atopic dermatitis compared to their matched healthy controls [12]. No prospective associations have been shown yet, so the direction of potential associations remains unclear. Trajectories from birth to adolescence tracking DNAm variation early in life have suggested poor performance of traditional clocks in children (Horvath [13] having been the first pan‐tissue epigenetic clock including mostly adult samples and Hannum [14] being accurate in blood but trained only on adults), which underlines the need for pediatric clocks [15, 16].

Therefore, we use the pediatric epigenetic clock recently developed by Wu et al. [17] to investigate associations between EAA and allergic diseases, namely asthma, allergic rhinitis, or eczema, as well as aeroallergen sensitization in the LISA birth cohort and validate results in the independent BAMSE birth cohort. We additionally investigate if genetic predisposition captured by polygenic risk scores (PRS) might drive these associations and if methylation risk scores (MRS) that have been associated with aeroallergen sensitization before [18] play a role. As EAA might also be relevant in allergic diseases during adulthood, which would raise the question of whether accelerated epigenetic age in childhood could potentially persist into adulthood, we perform additional analysis in a population‐based adult cohort as a first step in this direction. To the best of our knowledge, no studies on allergic phenotypes and EAA in adults exist so far. Further, we test the direction of associations using longitudinal data in LISA.

## 2 Methods

### 2.1 Study Population

Data from the prospective population‐based German birth cohort on the Influence of Life‐style factors on the Development of the Immune System and Allergies in East and West Germany (LISA) was used. Informed, written consent was obtained from the parents or legal guardians, and ethical approval was obtained from local ethics committees (Bavarian General Medical Council, Medical Council for North‐Rhine‐Westphalia, and the University of Leipzig). Further information is given elsewhere [19]. Our study included 240 children from the Munich study center with DNAm data available at the follow‐up of six (*N* = 234) and ten (*N* = 227) years of age.

### 2.2 Calculation of Epigenetic Age Metrics

DNAm was measured in blood using the MethylationEPIC BeadChip (Illumina Inc., San Diego, CA), as previously described [18]. DNA methylation age (DNAmAge) was calculated using the pediatric epigenetic clock developed by Wu et al. [17]. This methylation‐based age prediction model was trained on 716 healthy children (529 boys, 187 girls) between nine and 212 months old from 11 different DNAm datasets with either 27 K or 450 K methylation arrays. Data for four of the 111 CpG sites were not available on the EPIC chip; one was dropped during quality control in LISA. There was no recommendation by Wu et al. on how to handle missing CpGs, and no differentially methylated regions (DMR) were available for imputation. To shift DNAmAge values to a plausible range, the same arbitrary beta values were assigned for all children in such a way that the mean of predicted age equals the mean of chronological age. This was done separately for 6‐year‐olds and 10‐year‐olds without affecting association analysis results.

The residuals from regressing DNAmAge on chronological age in two linear models for 6 and 10 years, respectively, either adjusted (if not stated otherwise) or not adjusted for cell type proportions (CTPs) estimated by the EpiDISH method [20] were considered EAA and used for statistical analysis.

### 2.3 Allergy Phenotypes

The period prevalence of asthma, rhinitis, and eczema was determined at the 6‐ and 10‐year follow‐up from parent‐reported yearly physician diagnoses since the previous examination (for ages 5–6 years at the 6‐year follow‐up and ages 7–10 years at the 10‐year follow‐up). Any allergy was defined as the presence of at least one of these three diseases. Further, current asthma was defined as having at least two of the following three criteria: ever being diagnosed with asthma, having taken asthma medication within the past 12 months, or reporting wheezing within the last 12 months. Early eczema was defined as doctor diagnosis of eczema in the first 2 years of life. The presence of symptoms of allergic diseases within the last 12 months was asked at the 6‐ and 10‐year follow‐up, namely wheezing, nose symptoms (sneezing, runny or blocked nose) without having a cold accompanied by eye symptoms (itchy watery eyes) (rhinoconjunctivitis symptoms [21]), and skin rash at relevant regions of the body (bends of the elbows, back of the knees, wrists or ankles, face, neck) coming and going over at least 6 months. Aeroallergen sensitization was defined as specific IgE of > 0.35kU/L, measured using the CAP‐RAST FEIA system (Pharmacia Diagnostics, Freiburg, Germany) for a mix of common aeroallergens (SX1).

### 2.4 Allergy‐Related Polygenic and Methylation Risk Scores

As described previously [18, 22], PRS for asthma [23], allergic rhinitis [24], eczema [25], and any allergy [26], as well as allergy‐related MRS [18] based on high total IgE [4] and any allergy [7] were used to examine the role of genetic predisposition and MRS, considered an allergic disease biomarker, in EAA.

### 2.5 Statistical Analysis

Associations between the allergic phenotypes and EAA were estimated adjusting for time point (ages 6/10), sex, BMI, season of blood withdrawal (allergy season from March to August), parental education (high vs. medium/low), family atopy, and maternal smoking during pregnancy. To account for repeated measurements, linear mixed models were used to analyze the association of EAA as the outcome with the allergic phenotype as exposure, including a random intercept for individuals. Interactions between allergy phenotypes and sex as well as time point were tested.

Additionally, EAA (not adjusted for CTPs) was descriptively compared between three different groups representing allergic disease persistence: no allergy, any allergy at one timepoint, and any allergy at both timepoints. For this analysis, only children with available data at both time points were included. Further, EAA (not adjusted for CTPs) was compared by the number of allergies at each time point, representing allergic multimorbidity.

To disentangle the direction of effect, prospective linear and logistic regression analyses were conducted, including individuals with complete data at both time points (*n* = 196). The logistic model used any allergy at 10 years as the outcome and EAA at 6 years as the exposure. The linear models used EAA at 10 years as the outcome; as exposure, one used any allergy at 6 years, while the other one used a categorical variable with the categories no allergy, any allergy only at 6 years, any allergy only at 10 years, and any allergy at both time points. All models were adjusted for BMI, sex, parental education, and smoking during pregnancy. The logistic model was additionally adjusted for any allergy at 6 years and EAA at 10 years, and the linear model using any allergy at 6 years as exposure was additionally adjusted for EAA at 6 years.

### 2.6 Sensitivity Analysis

Associations of EAA and sensitization were additionally analyzed in children without asthma, rhinitis, or eczema diagnosis to investigate if associations might be conferred by non‐symptomatic sensitization. Associations of EAA (adjusted or not adjusted for CTPs) with allergy‐related PRS and MRS were examined. Further, analysis was performed using the Horvath skin&blood clock [16, 27] and Horvath pan‐tissue [13] clock, which both also included children in their training datasets. Intrinsic epigenetic age acceleration (IEAA) adjusting for CTPs was calculated for both clocks, applying the same approach for missing CpGs as for the Wu clock for comparability, assigning a beta value of maximal 1 to the methylation levels of missing CpG sites.

### 2.7 Replication in the BAMSE Birth Cohort

For replication, 625 children with allergy and DNAm data at the 8‐year follow‐up of the independent Swedish BAMSE birth cohort were included. Ethical approval for the study was given by the Regional Ethics Board (EPN); further information can be found elsewhere [28, 29]. DNAm data was measured with the Illumina Infinium HumanMethylation450 Bead‐Chip (Illumina Inc., San Diego, USA) [7] and the same allergy outcomes and confounder variables as in the LISA study were used. Information on a doctor's diagnosis of asthma, allergic rhinitis, or eczema within the last 12 months was collected as part of the 8‐year follow‐up questionnaire.

Results from LISA and BAMSE were meta‐analyzed applying inverse variance meta‐analysis with random effects.

### 2.8 Additional Analysis in the Adult KORA F4 Cohort

The KORA F4 cohort is a follow‐up of the fourth baseline survey of the Cooperation for Health Research in the Region of Augsburg (KORA S4). The study was approved by the Bavarian Medical Association Ethics Committee. Details can be found elsewhere [30]. In total, 1721 participants with available information on DNAm (measured with Illumina Infinium Human Methylation 450 K BeadChip) and the presence of doctor‐diagnosed asthma, hay fever, and eczema were included. The Horvath pan‐tissue [13] and skin&blood clock [13, 27] were used to calculate IEAA [31], as these clocks are widely used and have also been applied to pediatric populations. Linear models with IEAA as the outcome and allergic phenotype as exposure were adjusted for sex, BMI, smoking (never, ever, current), education (high vs. low/middle), physical activity (active vs. inactive), alcohol consumption (0 g/day, 0.1–39.9 g/day (men)/0.1–19.9 g/day (women), ≥ 40 g/day (men)/≥ 20 g/day (women)) [32], and cardiovascular disease (hypertension, myocardial infarction, or stroke).

All analyses were performed in R [33] V4.3.1 in LISA and KORA and V4.3.2 in BAMSE. The *lme4* [34] package V1.1.35.2 was used for linear mixed models, and the *meta* [35] package V7.0.0 for the meta‐analysis. Effect estimates are presented as absolute change in EAA together with 95% confidence intervals (95% CI). Correlations were stated using the Pearson or Spearman correlation coefficient.

## 3 Results

### 3.1 Study Participants

In total, 240 children from the LISA cohort were included, of which 234 had data available at the 6‐year follow‐up and 227 at the 10‐year follow‐up, with an overlap of 221. For replication, 625 children from the 8‐year follow‐up of BAMSE were included. Population characteristics are shown in Table 1.

**TABLE 1:** Description of the LISA and BAMSE study population.

	LISA		BAMSE
	Follow‐up at 6 years	Follow‐up at 10 years	Follow‐up at 8 years
	(*N* = 234)	(*N* = 227)	(*N* = 625)
	Mean (SD) or *n* (%) [*n* missings]	Mean (SD) or *n* (%) [*n* missings]	Mean (SD) or *n* (%) [*n* missings]
**Basic population characteristics**			
Age at DNAm measurement [years]	6.07 (0.151)	10.2 (0.136)	8.32 (0.480)
Child's BMI (kg/m^2^)	15.3 (1.27) [1]	17.0 (2.47) [1]	17.1 (1.93) [5]
Male sex	135 (57.7%)	131 (57.7%)	338 (54.1%)
High parental education (vs. low/middle)	186 (79.5%) [2]	181 (79.7%) [2]	314 (50.2%)
Blood taken in allergy season (march to august)	158 (67.5%)	116 (51.1%)	305 (48.8%)
Smoking during pregnancy	17 (7.3%) [7]	16 (7.0%) [6]	70 (11.2%)
Family atopy	151 (64.5%)	148 (65.2%)	216 (34.6%) [6]
**Allergic disease phenotypes**			
Doctor diagnosis^a^			
Asthma	6 (2.6%)	14 (6.2%) [10]	32 (5.1%)
Allergic rhinitis	13 (5.6%) [3]	42 (18.5%) [11]	19 (3.0%)
Eczema	25 (10.7%)	9 (4.0%) [9]	33 (5.3%)
Any allergy	38 (16.2%) [2]	51 (22.5%) [14]	68 (10.9%)
Other disease definitions			
Current asthma^b^	6 (2.6%) [1]	12 (5.3%) [3]	156 (25.0%)
Early eczema (within first 2 years of life)	43 (18.4%) [6]	40 (17.6%) [6]	145 (23.2%)
Symptoms			
Wheezing	25 (10.7%) [2]	18 (7.9%) [2]	130 (20.8%)
Rhinoconjunctivitis	20 (8.5%) [1]	34 (15.0%) [4]	66 (10.6%)
Skin rash	10 (4.3%) [4]	2 (0.9%) [6]	111 (17.8%)
Aeroallergen sensitization			
Including children with a diagnosed allergic disease	74 (31.6%)	101 (44.5%)	161 (25.8%)
Excluding children with a diagnosed allergic disease	48 (20.5%)	54 (23.8%)	120 (19.2%)

^a^ LISA: defined as having been diagnosed by a doctor since the last examination of the study (for ages 5 and 6 years at the 6‐year follow‐up and ages 7 to 10 years at the 10‐year follow‐up), BAMSE: defined as doctor diagnosis within the last 12 months.

^b^ Defined as having at least two of the following three criteria: ever being diagnosed with asthma, having taken asthma medication within the past 12 months, or reporting wheezing within the last 12 months.

In the adult KORA F4 cohort (*N* = 1721), participants had a mean age of 61.0 (±8.9) years (Table S1).

### 3.2 Performance of the Wu Clock in Comparison to Horvath Clocks

In LISA, DNAmAge based on the Wu clock was correlated with chronological age with *r* = 0.75; the median absolute error (MAE) was 0.04. The Horvath pan‐tissue clock reached a similar correlation (*r* = 0.74) and lower accuracy (MAE = 0.11), whereas the Horvath skin&blood clock showed a similar correlation (*r* = 0.77) as well, but even lower accuracy in estimating children's epigenetic age (MAE = −1.22) (Figure S1).

### 3.3 Associations of Epigenetic Age Acceleration With Allergic Diseases

**FIGURE 1:**
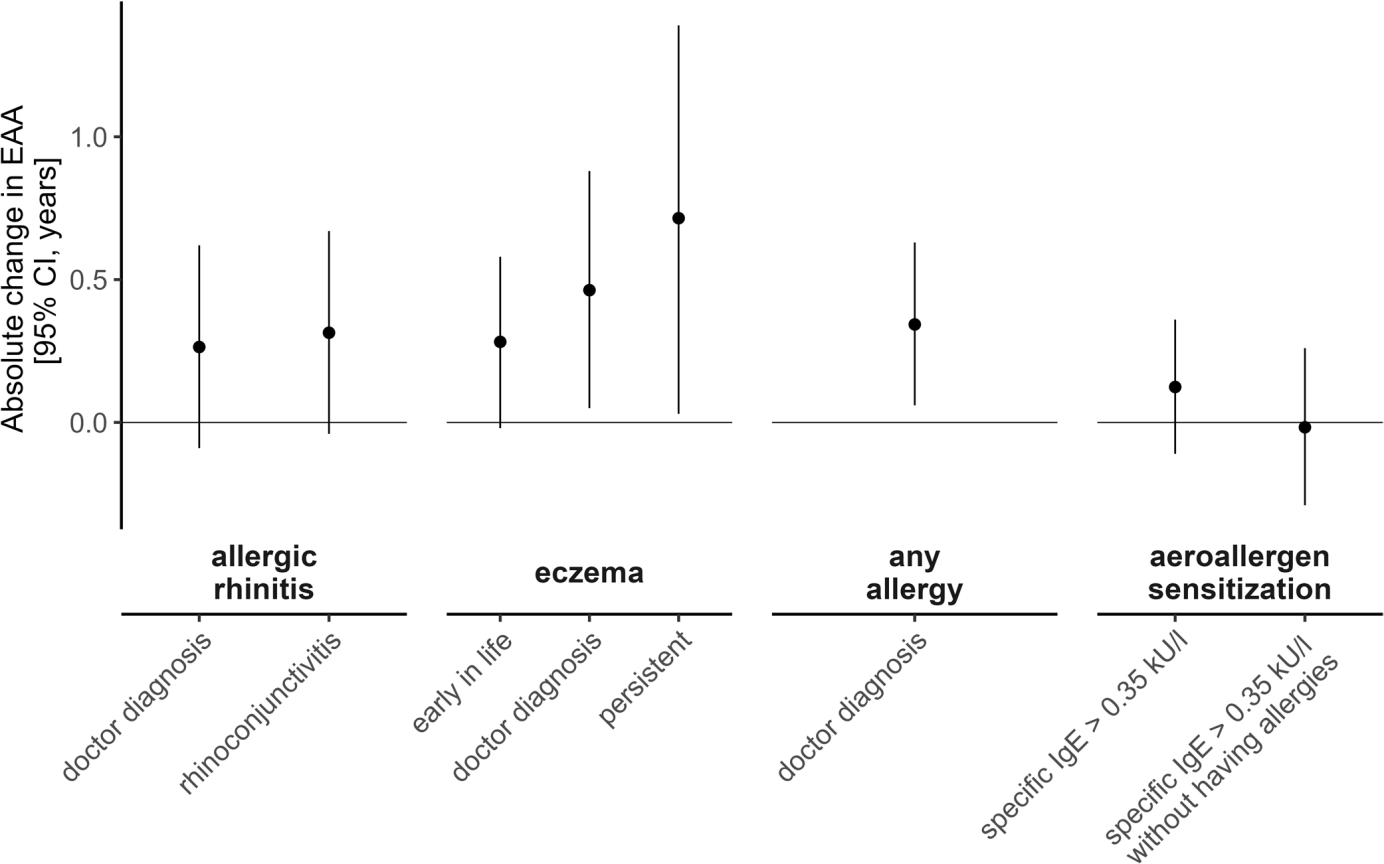
Cell type proportion adjusted epigenetic age acceleration (EAA) in children from the LISA birth cohort based on linear mixed models with the following phenotypes: Allergic rhinitis, eczema, eczema early in life, persistent eczema (early in life and current doctor diagnosis), rhinoconjunctivitis, any doctor‐diagnosed allergy and aeroallergen sensitization with and without having allergies.

In LISA, children with any allergy had an EAA increased by 0.34 years (95% CI = [0.06; 0.63]). Eczema was associated with a significantly higher EAA by 0.46 years (95% CI = [0.05; 0.88]). For rhinitis, a similar tendency was observed with an increased EAA of 0.26 years (95% CI = [−0.09; 0.62]). No associations with aeroallergen sensitization were observed (Figure 1, Table S2).

About 33% of 6‐year‐olds and 44% of 10‐year‐olds with eczema also had early eczema. Persistent disease (i.e., early and recent eczema) was significantly associated with EAA (0.72 years, 95% CI = [0.03; 1.39]), but early eczema was not (0.28 years, 95% CI = [−0.02; 0.58]) (Figure 1, Table S2).

Not adjusting for CTPs, results were consistent, showing that children with any allergy had an EAA of 0.36 years (95% CI = [0.06; 0.67]) (Figure S2).

Significant interactions with sex were observed for allergic rhinitis: boys having rhinitis were epigenetically 1 year older compared to girls with rhinitis (1.00 years, 95% CI = [0.26; 1.76]) (Figure S3). No significant interactions with sex were observed for the other phenotypes.

A significant interaction with follow‐up time point was observed only for early eczema: EAA significantly increased from the 6‐year to the 10‐year follow‐up (0.56 years, 95% CI = [0.04; 1.08]) (Figure S4).

### 3.4 Descriptive Comparison of EAA (Not Adjusted for CTPs) Regarding Allergic Disease Persistence and Allergic Multimorbidity

**FIGURE 2:**
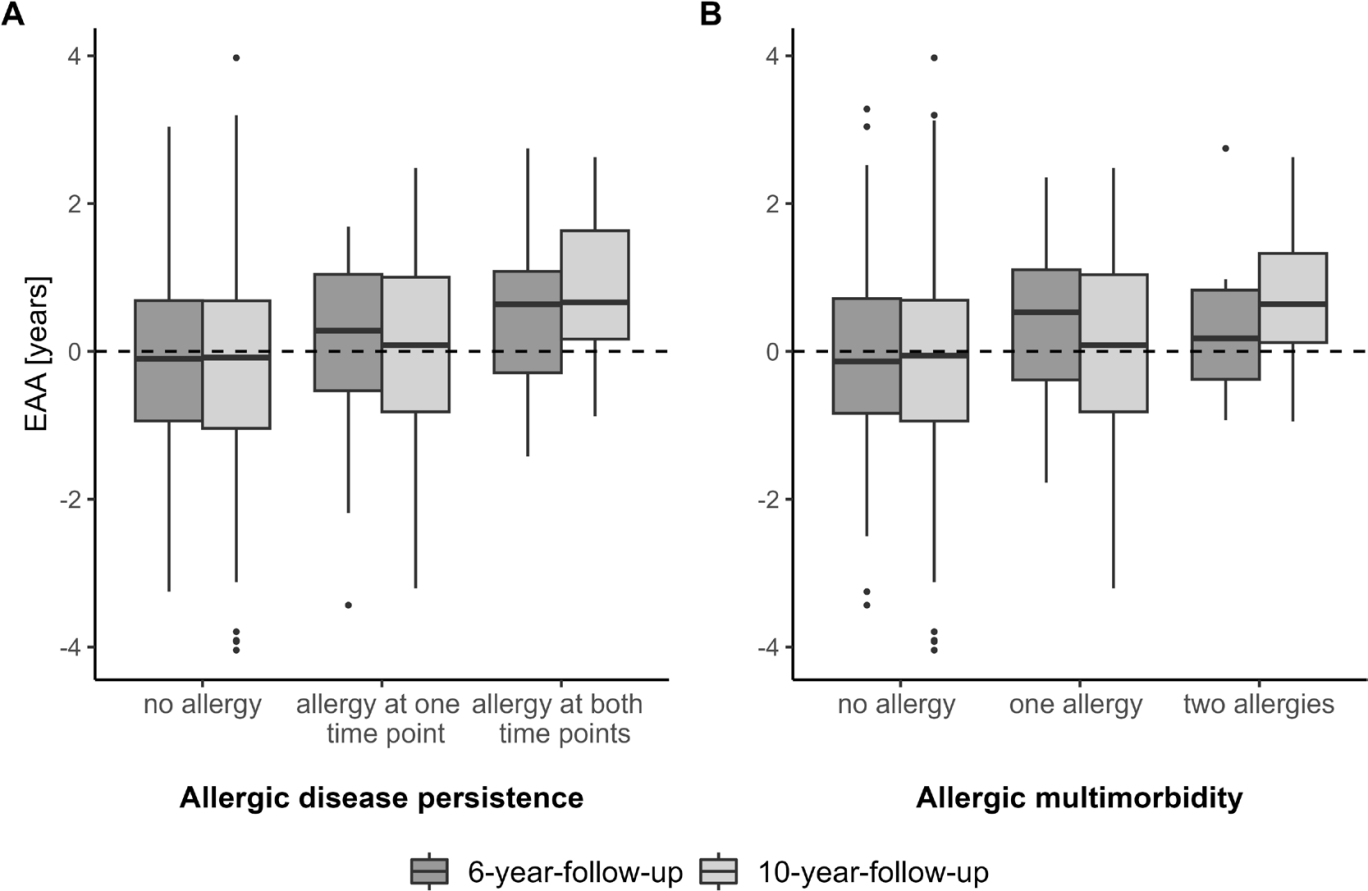
Descriptive comparison of the epigenetic age acceleration (EAA) (not adjusted for cell type proportions) distribution between (A) non‐allergic children, children with any allergy at the 6‐ or 10‐year follow‐up, and children with any allergy at the 6‐ and 10‐year follow‐up representing allergic disease persistence and (B) non‐allergic children, children with one allergic disease, and children with two allergic diseases representing allergic multimorbidity.

Children reporting any allergy at the 6‐ or 10‐year follow‐up had slightly higher EAA values compared to non‐allergic children, whereas the increase in EAA in children reporting persistent disease at both time points was more pronounced (Figure 2A). Further, for 10‐year‐olds, a gradient of EAA in response to allergic multimorbidity was observed, whereas for 6‐year‐olds, only an increase in EAA between non‐allergic children and children with one allergy was visible (Figure 2B).

### 3.5 Prospective Analysis

**FIGURE 3:**
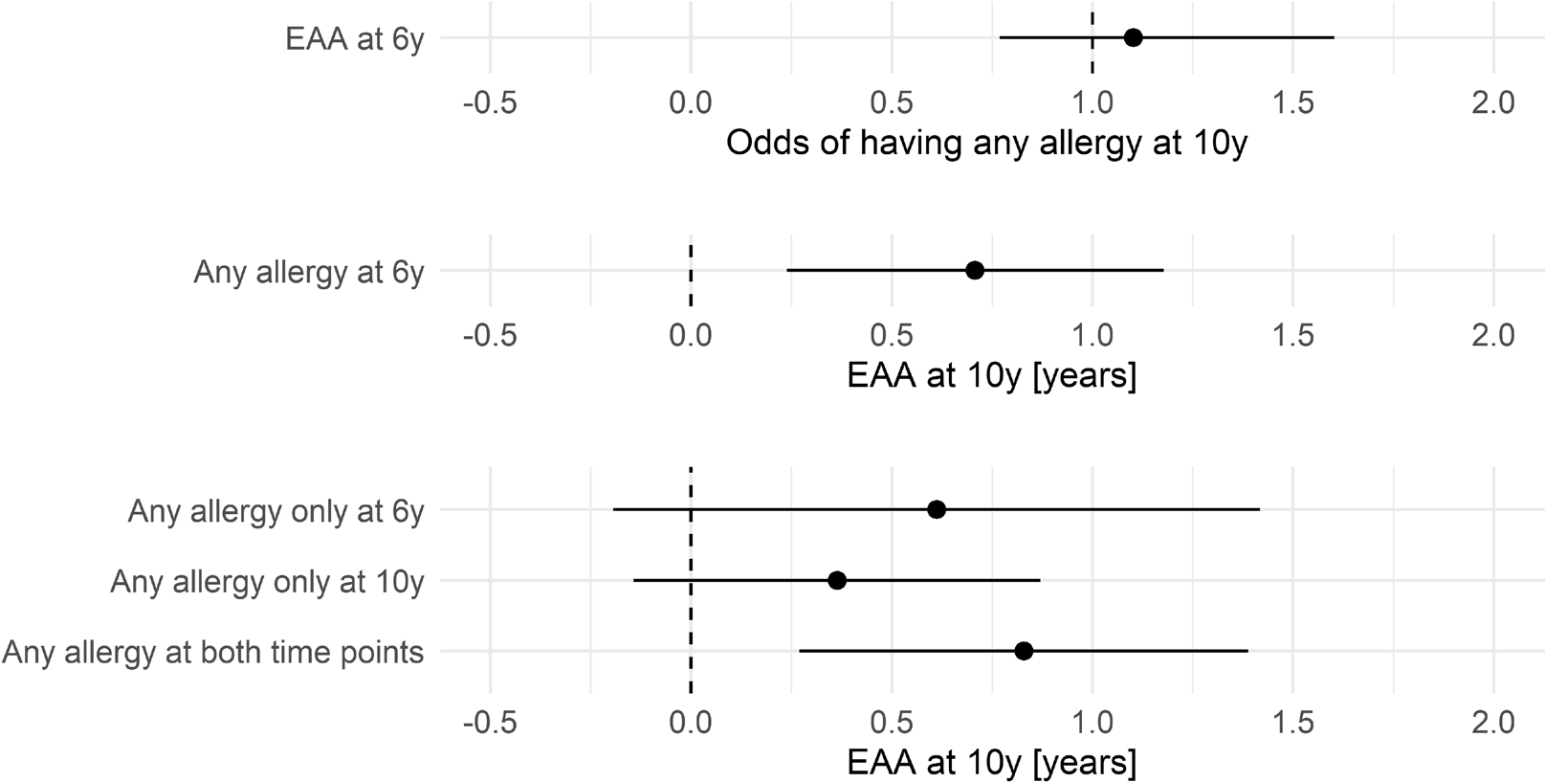
Prospective associations between cell type proportion adjusted epigenetic age acceleration (EAA) at 6 years and any allergy at 10 years, any allergy at 6 years and EAA at 10 years, and any allergy categories (any allergy only at 6 years, only at 10 years or at both time points) and EAA at 10 years.

In the prospective analysis, having any allergy at 6 years was significantly associated with an EAA increase at the 10‐year follow‐up by 0.70 years (95% CI = [0.24; 1.17]), while having any allergy at both time points was associated with a significant EAA increase at 10 years by 0.83 years (95% CI = [0.27; 1.39]). In contrast, no significant association was found between EAA at 6 years and having any allergy at 10 years (Figure 3).

### 3.6 Sensitivity Analyses

Despite the small number of asthma cases (*N* = 6 at age 6 years), results indicate an increase of EAA in asthmatic children (0.50 years, 95% CI = [−0.04; 1.04]) (Table S2).

A significant association was found for one MRS with EAA not adjusted for CTPs but not when adjusting for them, whereas for the PRS, no increase in both was observed (Figures S5 and S6, Table S2).

Using the Horvath pan‐tissue clock, rhinoconjunctivitis was associated with a decreased EAA (Figure S7), whereas all other allergic phenotypes did not show any significant associations in both Horvath clocks (Figures S7 and S9). However, both allergy‐related MRS were significantly associated with increased EAA when using the skin&blood clock and non‐significantly when using the pan‐tissue clock (Figures S8 and S10).

### 3.7 Replication in the BAMSE Birth Cohort

**FIGURE 4:**
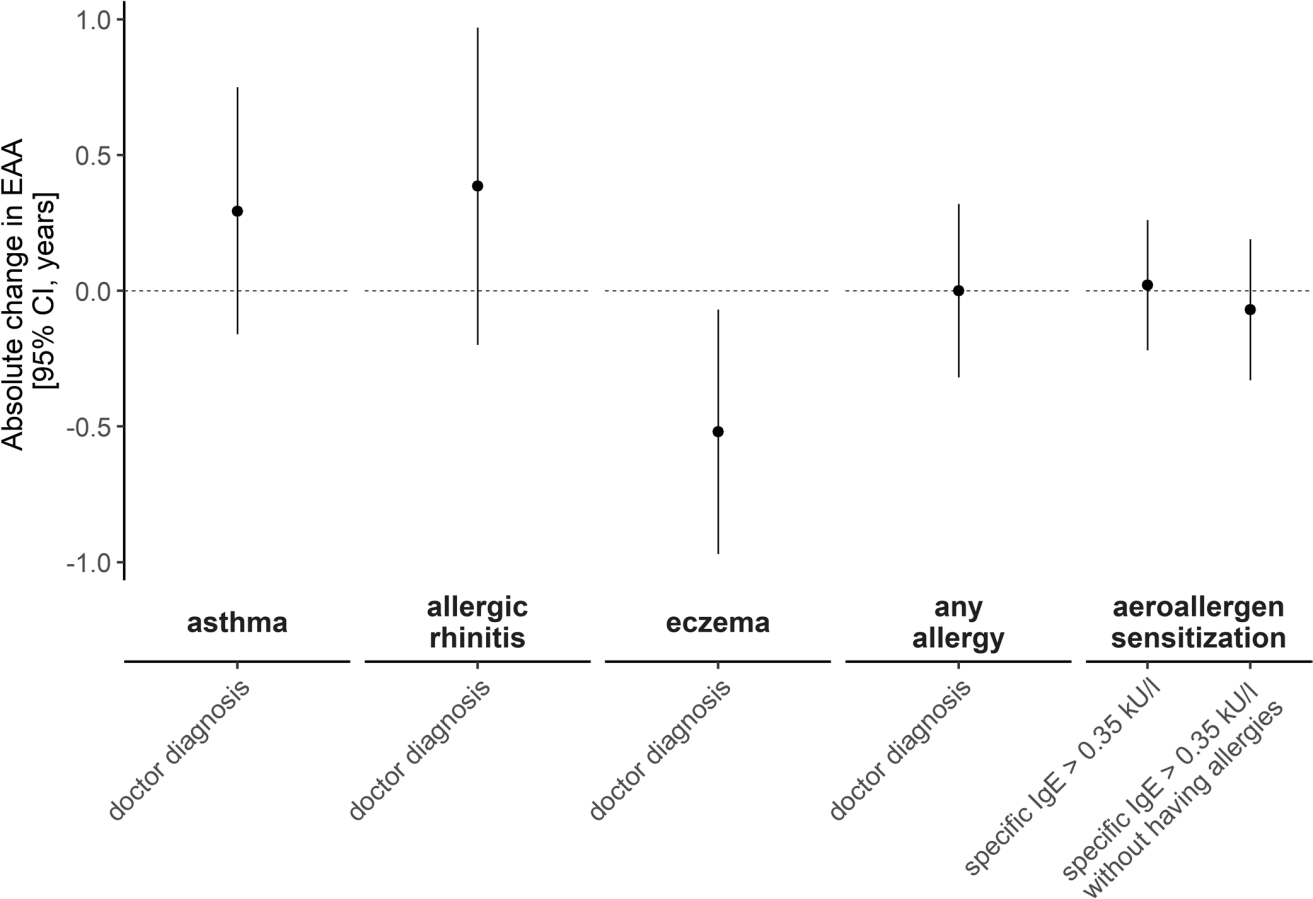
Cell type proportion adjusted epigenetic age acceleration (EAA) in children from the BAMSE birth cohort with doctor‐diagnosed asthma, allergic rhinitis, eczema, any allergy, or aeroallergen sensitization with and without having allergies.

In BAMSE, non‐significant positive associations with EAA were observed for asthma (0.29 years, 95% CI = [−0.16; 0.75]) and allergic rhinitis (0.39 years, 95% CI = [−0.2; 0.97]). However, current eczema was inversely associated with EAA (−0.52 years, 95% CI = [−0.97; −0.07]) (Figure 4, Table S2), with about 70% of those with current eczema also having had early eczema. No associations with aeroallergen sensitization or interactions with sex were observed. When meta‐analyzing results from LISA and BAMSE, the association with asthma became significant (Figure S11).

### 3.8 Additional Analysis in the Adult KORA F4 Cohort

**FIGURE 5:**
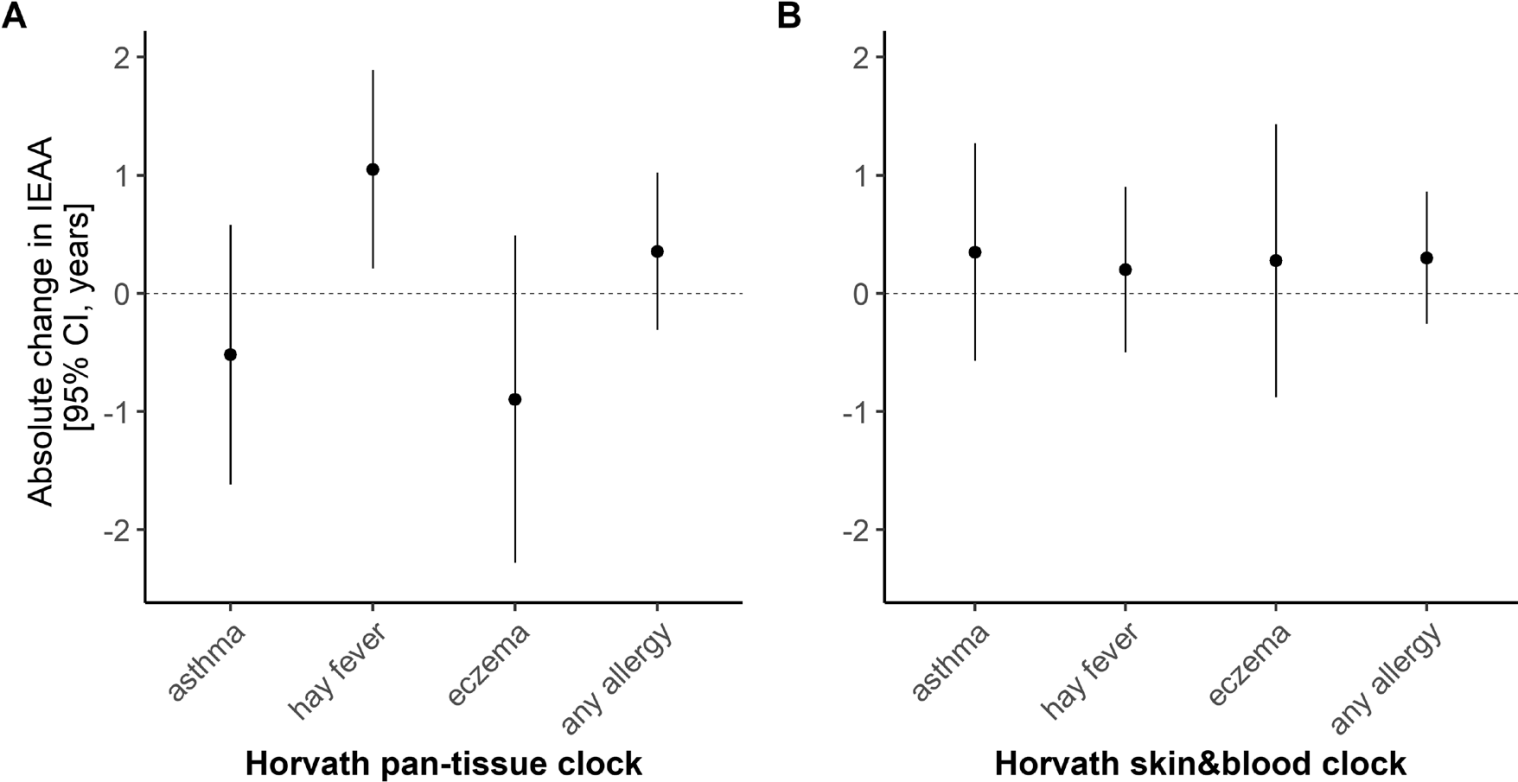
Intrinsic epigenetic age acceleration (IEAA) in adults from the KORA F4 cohort with doctor‐diagnosed asthma, hay fever, eczema, or any allergy. (A) Horvath pan‐tissue clock, (B) Horvath skin&blood clock.

The Horvath pan‐tissue and skin&blood clock were correlated with *r* = 0.77 in KORA F4 (Figure S12). Adults with hay fever had a significantly accelerated IEAA when using the Horvath pan‐tissue clock (1.05 years, 95% CI = [0.21; 1.89]) (Figure 5, Table S3), while this association was limited to males when using the Horvath skin&blood clock (1.25 years, 95% CI = [0.29; 3.15], Figure S13).

## 4 Discussion

In this study, we found an accelerated epigenetic age in children with allergic diseases from the LISA study, using the pediatric epigenetic clock by Wu et al. [17]. We observed similar but not statistically significant trends in children with asthma or allergic rhinitis from BAMSE, whereas an inverse association of EAA with eczema was found in BAMSE. Adults with hay fever from KORA F4 had a significantly increased EAA when using the Horvath skin&blood clock.

In line with our results, Peng et al. [10] demonstrated cross‐sectional associations of EAA with higher odds for having asthma at mid‐childhood using the Horvath pan‐tissue clock. Also consistent with our results, the study from Cardenas et al. using the Horvath pan‐tissue clock found a significant EAA in nasal tissue from asthmatic children [13], with a slightly higher effect size compared to our results. Additionally, Peng et al. observed significant associations between age acceleration and aeroallergen sensitization, while we did not, using a slightly different allergen composition for aeroallergen sensitization. Our results suggest that the associations with accelerated epigenetic age might be stronger in those with persistent allergic disease burden, and not conferred by non‐symptomatic sensitization. Beyond previous studies, we found a significantly increased IEAA in adults and predominantly men with hay fever. In children, the association with rhinitis was limited to males. Jeremian et al. recently found accelerated epigenetic age in two‐year‐old children with atopic dermatitis using a case–control design [12]. Our results from LISA support these findings while covering another age group and early eczema as an additional phenotype, using a population‐based approach. Interestingly, in BAMSE, we observed an inverse association of current eczema with EAA. As in BAMSE, the age of onset of eczema was early in life for most children, it might be speculated that this association could potentially be explained by a greater health awareness of parents when their child is affected by early eczema, providing a healthier lifestyle and environment to those children, resulting in a decelerated epigenetic age. In addition, parents of children with a known eczema history might seek medical assistance earlier, resulting in better disease control and management. However, this does not fully explain the opposing findings in LISA and BAMSE. We can only assume that this is due to unassessed differences in lifestyle or environmental factors, or eczema severity, and a high contribution of individual observations in this specific phenotype due to relatively low case numbers. The finding of early eczema being associated with increased EAA only at the 10‐year follow‐up suggests a persistent effect which might also result from an interaction with unmeasured environmental exposures or other factors. However, case numbers are too small to further disentangle this within this study.

We could not replicate our results in clocks mainly trained on adults like the Horvath skin&blood and pan‐tissue clock, the latter showing partly inconsistent results. This might be explained by the fact that these clocks were less precise in predicting children's chronological age lying within a very narrow age range. In addition, they are based on methylation sites mainly relevant for epigenetic aging in adults and might therefore not be involved in childhood allergic diseases.

Biological mechanisms underlying the observed associations remain mostly unexplained. It can be speculated that one mechanism might be increased immune cell activities accompanied by chronic inflammation during allergic events leading to stress and accelerated aging of those cells. An allergen challenge followed by pro‐inflammatory cytokine emission can lead to increased production of effector cells such as basophils or eosinophils by stimulating the differentiation of hematopoietic progenitor cells [36]. Hematopoietic stem or progenitor cells (HSPCs) are for several reasons suggested to be involved in asthma and allergic diseases, e.g., in asthmatic patients CD34+ progenitor cells (HSPCs) were elevated in the sputum following exposure to an inhaled allergen [37]. So, it has been suggested that HSPCs show an increased migratory response in the context of allergic inflammation [36, 38]. It would be of great interest to investigate if HSPCs also age faster when producing more effector cells during allergic reactions.

To the best of our knowledge, all existing studies have been cross‐sectional or did not find any significant prospective associations [10]. Therefore, the direction of the observed associations remains unclear. Peng et al. argue that age acceleration might rather lead to a higher risk of allergy and asthma than being a consequence of it. However, our results could be interpreted as a hint toward the hypothesis that epigenetic age is accelerated due to being affected by allergic diseases because of the following observations: (1) Prospective associations were not observed between EAA at 6 years and any allergy at 10 years, but in the opposite direction. The latter might be driven by a cross‐sectional effect through any allergy at 10 years. Yet, results from the model considering any allergy categories based on both time points as exposure hint toward a more pronounced association with EAA at 10 years in those having any allergy only at 6 years or at both time points. Though, confidence intervals are large reflecting low sample size. (2) Aeroallergen sensitization did not show significant associations. However, sensitization by the initial exposure to an allergen is a prerequisite for the development of allergic symptoms following subsequent exposures [39]. So, if accelerated epigenetic aging led to increased allergic disease susceptibility, we would also expect associations with sensitization. (3) Allergy‐related MRS were partly directly associated with EAA. The MRS are based on EWAS of IgE or any allergy and can be seen as allergic disease surrogates [18]. Thus, a high MRS represents a high likelihood of ongoing allergic disease and also captures undiagnosed cases. This again indicates that it seems to be rather ongoing disease driving the association than increased susceptibility, e.g., due to genetic predisposition captured by PRS. Beyond that, our findings are supported by an increase of EAA in response to allergic disease persistence and allergic multimorbidity, indicating a stronger acceleration of epigenetic age in likely more severe cases. However, the latter aspect could also argue for EAA as a mechanism mediating the risk for allergic disease persistence and multimorbidity. This would be in line with previous work based on the same data suggesting single CpG sites as early predictors of allergic sensitization, mediating the effects of early‐life exposures [40]. As early‐life risk factors for allergic diseases have been shown to be associated with EAA [41], more research is needed to disentangle the direction of these associations. As proposed by Kilanowski et al., there might also be effects in both directions, depending on the genomic location, potentially both covered by the epigenetic clock [40].

This and previous studies suggest that EAA might be a sensitive biomarker of allergic diseases in childhood. This implicates the potential for improving early detection of individuals with high risk and assessing the severity of disease to better differentiate mild and severe cases in the future. Additionally, our results unmask novel implications of allergic diseases on children's health.

This study has several limitations. Due to the relatively low sample size, the statistical power to detect smaller effects for single allergic diseases is limited, and the possibilities to build subgroups of allergic disease phenotypes based on their manifestation age are restricted. The number of asthma cases was very small in LISA, thus limiting meaningful inference. However, there was a similar but non‐significant trend in the same direction in BAMSE, which holds a considerably higher number of asthma cases. To break down the intriguing eczema finding in BAMSE and identify any potential residual confounding, a better longitudinal resolution would be necessary. However, this is beyond the scope of this study and needs further research. The Wu clock was developed based on the 450 K methylation array from which few CpG sites were not available in LISA. Assuming the same beta values of the missing CpG sites for all participants to shift DNAmAge to a plausible range might alter the precision of the clock. However, this could solely have biased the results toward the null. The longitudinal birth cohort design is one of the strengths of this study. For a large proportion of children, data at two relevant time points during childhood were available, improving the reliability and generalizability of the findings. Further, this enabled prospective analyses which support results from linear mixed models. In comparison to existing studies mostly using the Horvath or other more established clocks, we used the pediatric clock by Wu et al. that was shown to have a higher accuracy in estimating children's epigenetic age [16, 17]. Other strengths are that extensive allergy definitions including symptoms were used and genetic data was available.

In summary, we found accelerated epigenetic age in children with any allergy, but not for aeroallergen sensitization alone in LISA. This study confirms findings from previous studies and adds evidence by using longitudinal data in combination with extensive allergic disease phenotypes and the use of a pediatric epigenetic clock. For future research, larger longitudinal studies and a life course approach would help to gain more insights into the role of EAA in allergic diseases.

## Author Contributions

M.L. conducted the analyses and involved in methodology, visualization, writing – original draft preparation, and writing – review and editing. E.T. contributed to conceptualization, methodology, supervision, and writing – review and editing. Z.Y. conducted formal analysis for validation and involved in writing – review and editing. A.H. contributed to conceptualization and writing – review and editing. Y.Y. performed epigenetic age calculation in KORA and contributed to writing – review and editing. S.K.M., O.G., and S.W. involved in data curation and writing – review and editing. M.W., E.M., and A.P. contributed to conceptualization, methodology, data curation, and writing – review and editing. M.S. supervised the project performing conceptualization, data curation, funding acquisition, methodology, and writing – review and editing. All the authors revised and commented on the final manuscript version.

## Conflicts of Interest

The authors declare no conflicts of interest.

## Supporting information


**Table S1.** Description of the KORA F4 study population.
**Table S2.** Epigenetic age acceleration (EAA) association analysis results in LISA and BAMSE using the Wu clock: Effect estimates, *p*‐values, and 95% confidence interval (CI) of main and other allergic phenotypes, polygenic risk scores (PRS) for allergic diseases and methylation risk scores (MRS) for high total IgE and any allergy.
**Table S3.** Intrinsic epigenetic age acceleration (IEAA) association analysis results in KORA F4 using the Horvath pan‐tissue and skin&blood clock: Effect estimates, *p*‐values, and 95% confidence interval (CI) of allergic phenotypes.
**Figure S1.** Correlations between DNAmAge and chronological age: (A) Wu clock: Spearman correlation coefficient *r* = 0.75, median absolute error (MAE) = 0.04 years, (B) Horvath pan‐tissue clock: *r* = 0.74, MAE = 0.11, (C) Horvath skin&blood clock: *r* = 0.77, MAE = −1.22.
**Figure S2.** Epigenetic age acceleration (EAA) (not adjusted for cell type proportions) in LISA children with asthma, allergic rhinitis, eczema, any allergy or aeroallergen sensitization.
**Figure S3.** Descriptive comparison of the cell type proportion adjusted epigenetic age acceleration (EAA) distribution in boys and girls from LISA with or without allergic rhinitis. The sex interaction term reached statistical significance (1.0 years, 95% CI [0.26; 1.76]).
**Figure S4.** Linear mixed model means of EAA in LISA at the 6‐ and 10‐year follow‐up for main allergic phenotypes, including time interaction terms. *There is a significant interaction effect between allergic phenotype and follow‐up time point.
**Figure S5.** Associations of cell type proportion adjusted epigenetic age acceleration (EAA) in LISA with polygenic risk scores (PRS) for asthma, rhinitis, eczema, and any allergy and methylation risk scores (MRS) for IgE and any allergy.
**Figure S6.** Associations of epigenetic age acceleration (EAA) (not adjusted for cell type proportions) with polygenic risk scores (PRS) for asthma, rhinitis, eczema, and any allergy and methylation risk scores (MRS) for IgE and any allergy.
**Figure S7.** Intrinsic epigenetic age acceleration (IEAA) calculated by the Horvath pan‐tissue clock in LISA children with allergic rhinitis, eczema, any allergy or aeroallergen sensitization.
**Figure S8.** Associations of intrinsic epigenetic age acceleration (IEAA) in LISA calculated by the Horvath pan‐tissue clock with polygenic risk scores (PRS) for asthma, rhinitis, eczema, and any allergy and methylation risk scores (MRS) for IgE and any allergy.
**Figure S9.** Intrinsic epigenetic age acceleration (IEAA) calculated by the Horvath skin&blood clock in LISA children with asthma, allergic rhinitis, eczema, any allergy or aeroallergen sensitization.
**Figure S10.** Associations of intrinsic epigenetic age acceleration (IEAA) in LISA calculated by the Horvath skin&blood clock with polygenic risk scores (PRS) for asthma, rhinitis, eczema, and any allergy and methylation risk scores (MRS) for IgE and any allergy.
**Figure S11.** Random effects inverse variance meta‐analysis of LISA and BAMSE results.
**Figure S12.** Correlation between the Horvath pan‐tissue (*y* axis) and Horvath skin&blood clock (x axis) within the KORA F4 cohort. The blue line represents a linear regression line. Pearson correlation coefficient *r* = 0.77.
**Figure S13.** Descriptive comparison of epigenetic age acceleration (EAA) distribution in males (light gray) and females (dark gray) with or without hay fever in adults from KORA F4, (A) using the Horvath pan‐tissue clock, and (B) the Horvath skin&blood clock. The interaction analysis revealed that men with hay fever were 1.72 years (95% CI [0.29; 3.15]) older than women with hay fever when using the Horvath skin&blood clock (B). When using the Horvath pan‐tissue clock (A), the interaction term did not reach statistical significance (0.65 years, 95% CI [−1.07; 2.36]).

## Data Availability

The data that support the findings of this study are available on request from the corresponding author. The data are not publicly available due to privacy or ethical restrictions.
