# Supplementary material for: Childhood Asthma and Allergy Are Related to Accelerated Epigenetic Aging
Source: Allergy. 2025 May 10;80(7):1912–22. doi: 10.1111/all.16583 (PMC12261877; doi:10.1111/all.16583)
Supplement: Supplementary file 1 — Table S1. Description of the KORA F4 study population. Table S2. Epigenetic age acceleration (EAA) association analysis results in LISA and BAMSE using the Wu clock: Effect estimates, p‐values, and 95% confidence interval (CI) of main and other allergic phenotypes, polygenic risk scores (PRS) for allergic diseases and methylation risk scores (MRS) for high total IgE and any allergy. Table S3. Intrinsic epigenetic age acceleration (IEAA) association analysis results in KORA F4 using the Horvath pan‐tissue and skin&blood clock: Effect estimates, p‐values, and 95% confidence interval (CI) of allergic phenotypes. Figure S1. Correlations between DNAmAge and chronological age: (A) Wu clock: Spearman correlation coefficient r = 0.75, median absolute error (MAE) = 0.04 years, (B) Horvath pan‐tissue clock: r = 0.74, MAE = 0.11, (C) Horvath skin&blood clock: r = 0.77, MAE = −1.22. Figure S2. Epigenetic age acceleration (EAA) (not adjusted for cell type proportions) in LISA children with asthma, allergic rhinitis, eczema, any allergy or aeroallergen sensitization. Figure S3. Descriptive comparison of the cell type proportion adjusted epigenetic age acceleration (EAA) distribution in boys and girls from LISA with or without allergic rhinitis. The sex interaction term reached statistical significance (1.0 years, 95% CI [0.26; 1.76]). Figure S4. Linear mixed model means of EAA in LISA at the 6‐ and 10‐year follow‐up for main allergic phenotypes, including time interaction terms. *There is a significant interaction effect between allergic phenotype and follow‐up time point. Figure S5. Associations of cell type proportion adjusted epigenetic age acceleration (EAA) in LISA with polygenic risk scores (PRS) for asthma, rhinitis, eczema, and any allergy and methylation risk scores (MRS) for IgE and any allergy. Figure S6. Associations of epigenetic age acceleration (EAA) (not adjusted for cell type proportions) with polygenic risk scores (PRS) for asthma, rhinitis, eczema, and any alle [file ALL-80-1912-s001.docx]

Supplement

Table S1: Description of the KORA F4 study population.

|  | **KORA F4 cohort** |
| --- | --- |
|  | (N=1721) |
|  | **mean (sd) or n (%)  [n missings]** |
| ***Basic population characteristics*** |  |
| Age at DNA methylation measurement [years] | 61.0 (8.89) |
| BMI (kg/m^2^) | 28.1 (4.78) [6] |
| Male sex | 839 (48.8%) |
| High education | 988 (57.4%) [3] |
| Smoking |  |
| Never Smoker | 721 (41.9%) |
| Former Smoker | 753 (43.8%) |
| Current Smoker | 247 (14.4%) |
| Family atopy | 282 (16.4%) [345] |
| Physical activity | 984 (57.2%) |
| Alcohol consumption |  |
| No | 509 (29.6%) |
| Low | 868 (50.4%) |
| Medium | 344 (20.0%) |
| Cardiovascular disease (hypertension/myocardial infarction/stroke) | 812 (47.2%) [2] |
| ***Allergic disease phenotypes*** |  |
| **Prevalence (doctor diagnosis within the last 12 months)** |  |
| Asthma | 81 (4.7%) [1] |
| Hay fever | 146 (8.5%) [1] |
| Eczema | 50 (2.9%) |
| Any allergy | 246 (14.3%) [2] |

Table S2: Epigenetic age acceleration (EAA) association analysis results in LISA and BAMSE using the Wu clock: Effect estimates, p-values, and 95 % confidence interval (CI) of main and other allergic phenotypes, polygenic risk scores (PRS) for allergic diseases and methylation risk scores (MRS) for high total IgE and any allergy.

|  | **Beta Estimate** | **p-value** | **95 % CI** |
| --- | --- | --- | --- |
| **LISA** |  |  |  |
| **Main allergic phenotypes** |  |  |  |
| Asthma | 0.500 | 0.072 | [-0.04; 1.04] |
| Rhinitis | 0.264 | 0.146 | [-0.09; 0.62] |
| Eczema | 0.463 | 0.031 | [0.05; 0.88] |
| Any allergy | 0.343 | 0.019 | [0.06; 0.63] |
| Aeroallergen sensitization | 0.124 | 0.314 | [-0.11; 0.36] |
| Aeroallergen sensitization without having allergies | -0.017 | 0.907 | [-0.29; 0.26] |
|  |  |  |  |
| **Other allergic phenotypes** |  |  |  |
| Current asthma | 0.241 | 0.419 | [-0.34; 0.83] |
| Early eczema | 0.282 | 0.070 | [-0.02; 0.58] |
| Wheezing | 0.293 | 0.140 | [-0.09; 0.69] |
| Rhinoconjunctivitis | 0.314 | 0.082 | [-0.04; 0.67] |
| Skin rash | 0.070 | 0.847 | [-0.63; 0.77] |
|  |  |  |  |
| **Polygenic risk scores (PRS)** |  |  |  |
| Asthma PRS | -0.025 | 0.675 | [-0.14; 0.09] |
| Rhinitis PRS | -0.015 | 0.817 | [-0.14; 0.11] |
| Eczema PRS | 0.094 | 0.132 | [-0.03; 0.21] |
| Any allergy PRS | 0.036 | 0.591 | [-0.09; 0.16] |
|  |  |  |  |
| **Methylation risk scores (MRS)** |  |  |  |
| Chen (IgE) | 0.032 | 0.610 | [-0.09; 0.16] |
| Xu (any allergy) | 0.061 | 0.413 | [-0.08; 0.21] |
|  |  |  |  |
| **BAMSE** |  |  |  |
| **Main allergic phenotypes** |  |  |  |
| Asthma | 0.293 | 0.208 | [-0.16; 0.75] |
| Rhinitis | 0.386 | 0.194 | [-0.2; 0.97] |
| Eczema | -0.519 | 0.023 | [-0.97; -0.07] |
| Any allergy | 0.000 | 0.999 | [-0.32; 0.32] |
| Aeroallergen sensitization | 0.021 | 0.864 | [-0.22; 0.26] |
| Aeroallergen sensitization without having allergies | 0.07 | 0.599 | [-0.33; 0.19] |

Table S3: Intrinsic epigenetic age acceleration (IEAA) association analysis results in KORA F4 using the Horvath pan-tissue and skin&blood clock: Effect estimates, p-values, and 95 % confidence interval (CI) of allergic phenotypes.

|  | **Beta Estimate** | **p-value** | **95 % CI** |
| --- | --- | --- | --- |
| **KORA F4** |  |  |  |
| **Horvath pan-tissue clock** |  |  |  |
| Asthma | -0.517 | 0.357 | [-1.62; 0.58] |
| Hay fever | 1.047 | 0.015 | [0.21; 1.89] |
| Eczema | -0.896 | 0.204 | [-2.28; 0.49] |
| Any allergy | 0.355 | 0.296 | [-0.31; 1.02] |
|  |  |  |  |
| **Horvath skin&blood clock** |  |  |  |
| Asthma | 0.348 | 0.458 | [-0.57; 1.27] |
| Hay fever | 0.201 | 0.574 | [-0.50; 0.90] |
| Eczema | 0.276 | 0.638 | [-0.88; 1.43] |
| Any allergy | 0.300 | 0.290 | [-0.26; 0.86] |


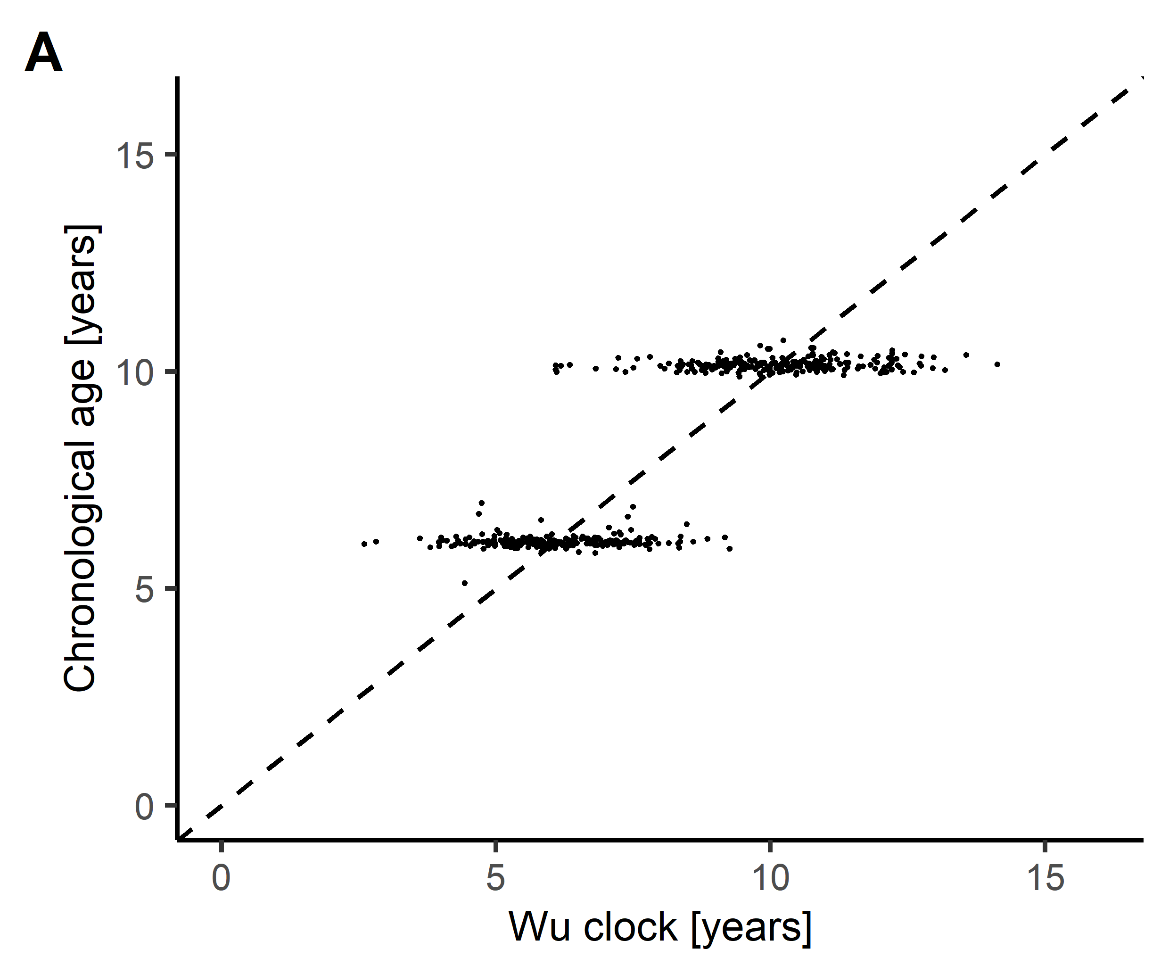


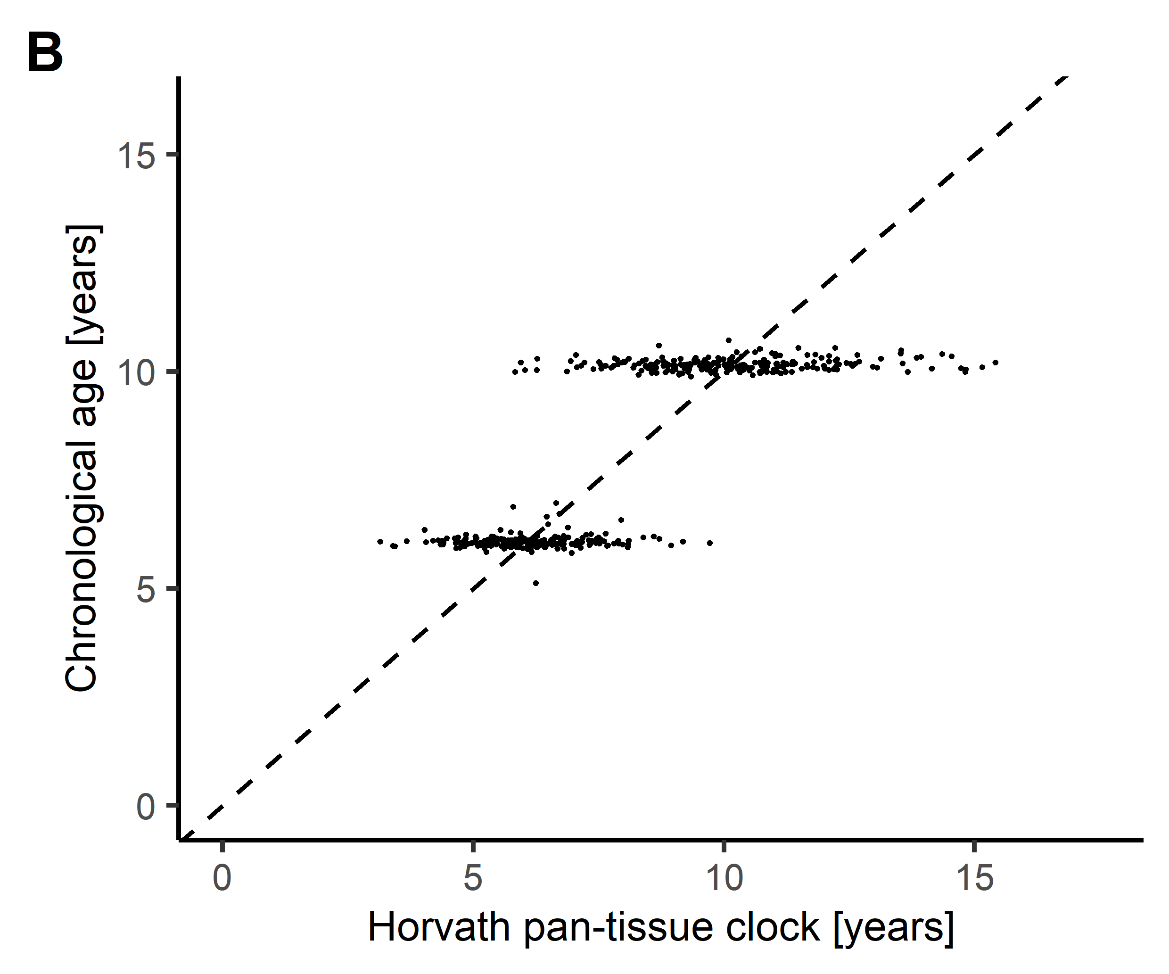


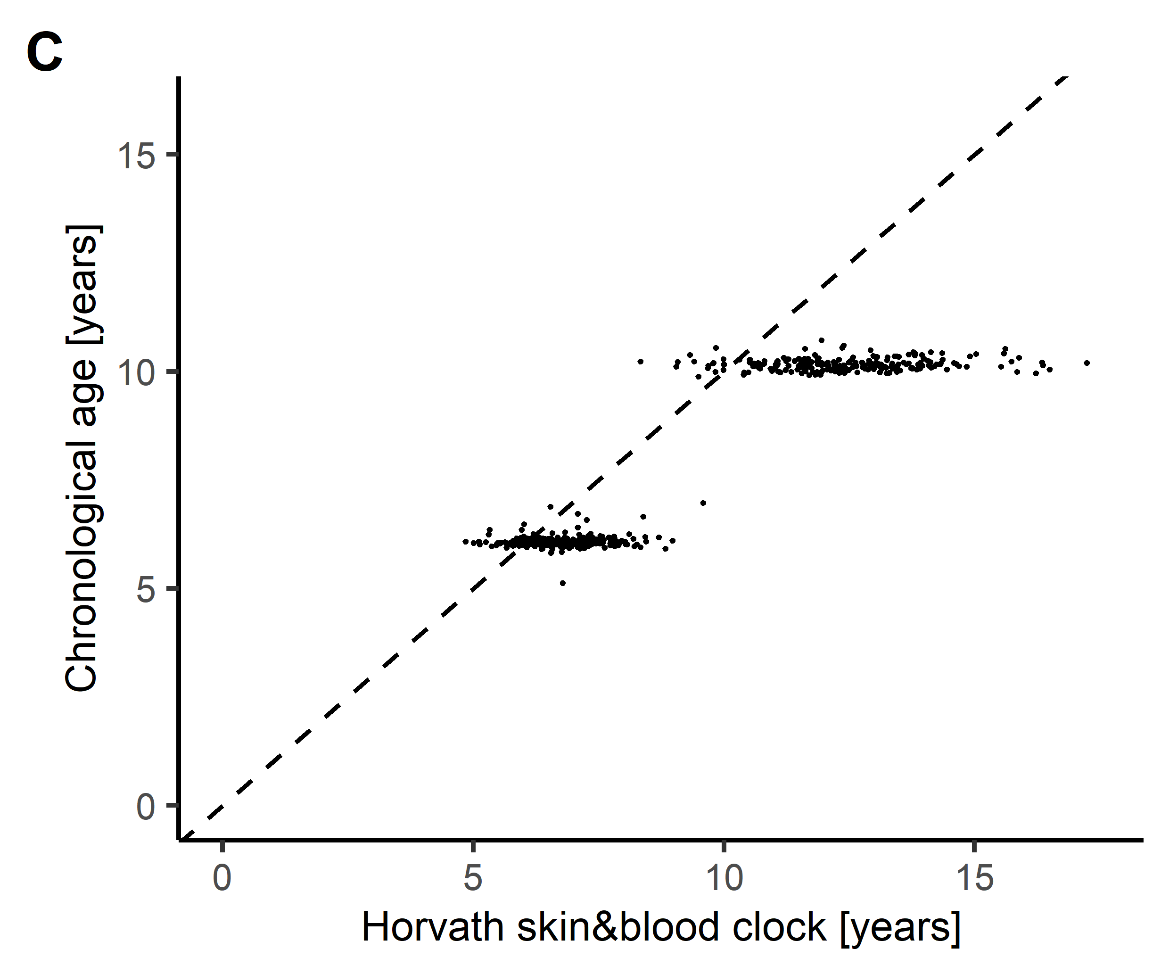


Figure S1: Correlations between DNAmAge and chronological age: (A) Wu clock: Spearman correlation coefficient r=0.75, median absolute error (MAE)=0.04 years, (B) Horvath pan-tissue clock: r=0.74, MAE=0.11, (C) Horvath skin&blood clock: r=0.77, MAE=-1.22. The black dashed line represents the perfect correlation between clock and chronological age.


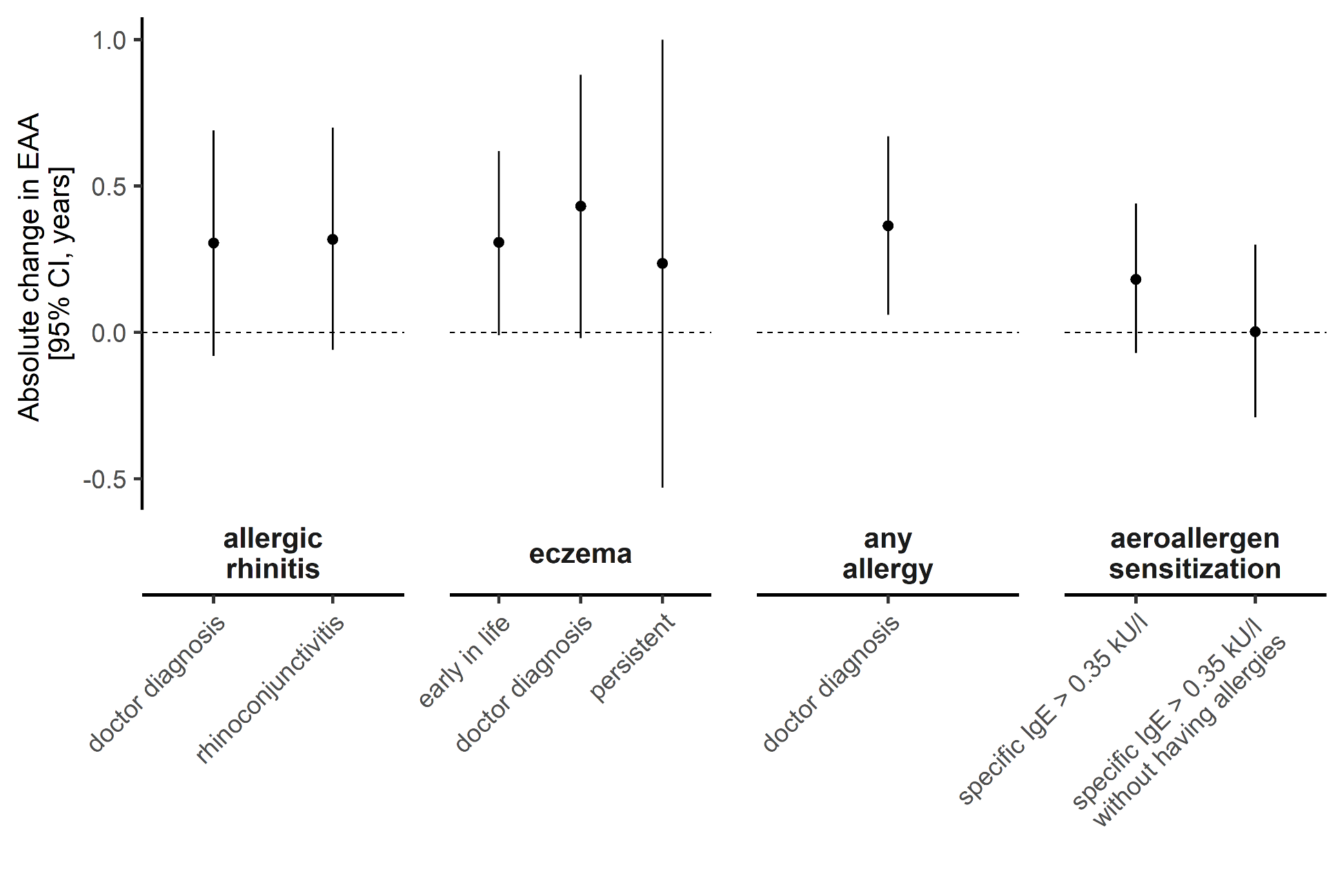


Figure S2: Epigenetic age acceleration (EAA) (not adjusted for cell type proportions) in LISA children with asthma, allergic rhinitis, eczema, any allergy or aeroallergen sensitization.


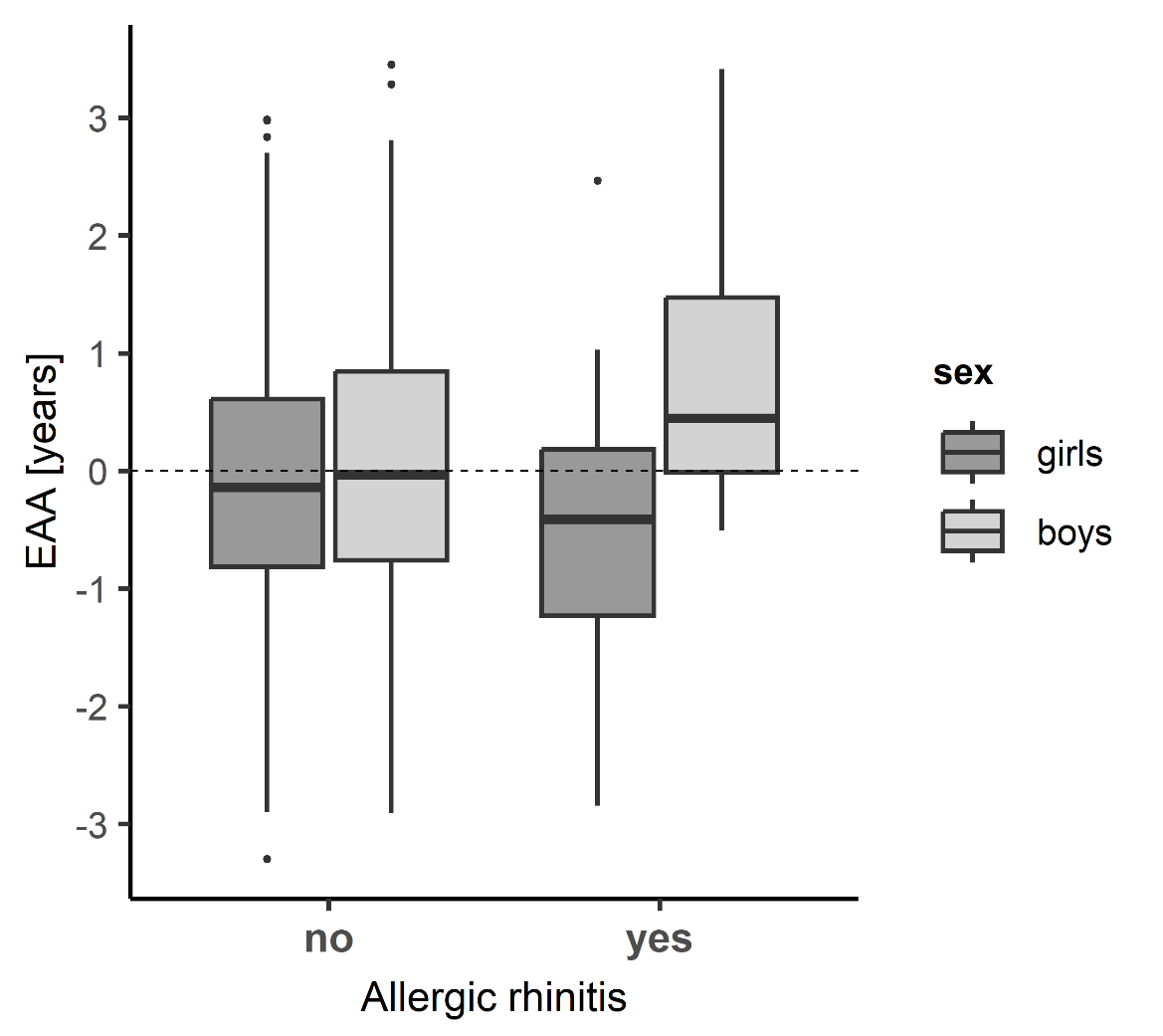


Figure S3: Descriptive comparison of the cell type proportion adjusted epigenetic age acceleration (EAA) distribution in boys and girls from LISA with or without allergic rhinitis. The sex interaction term reached statistical significance (1.0 years, 95%CI [0.26;1.76]).


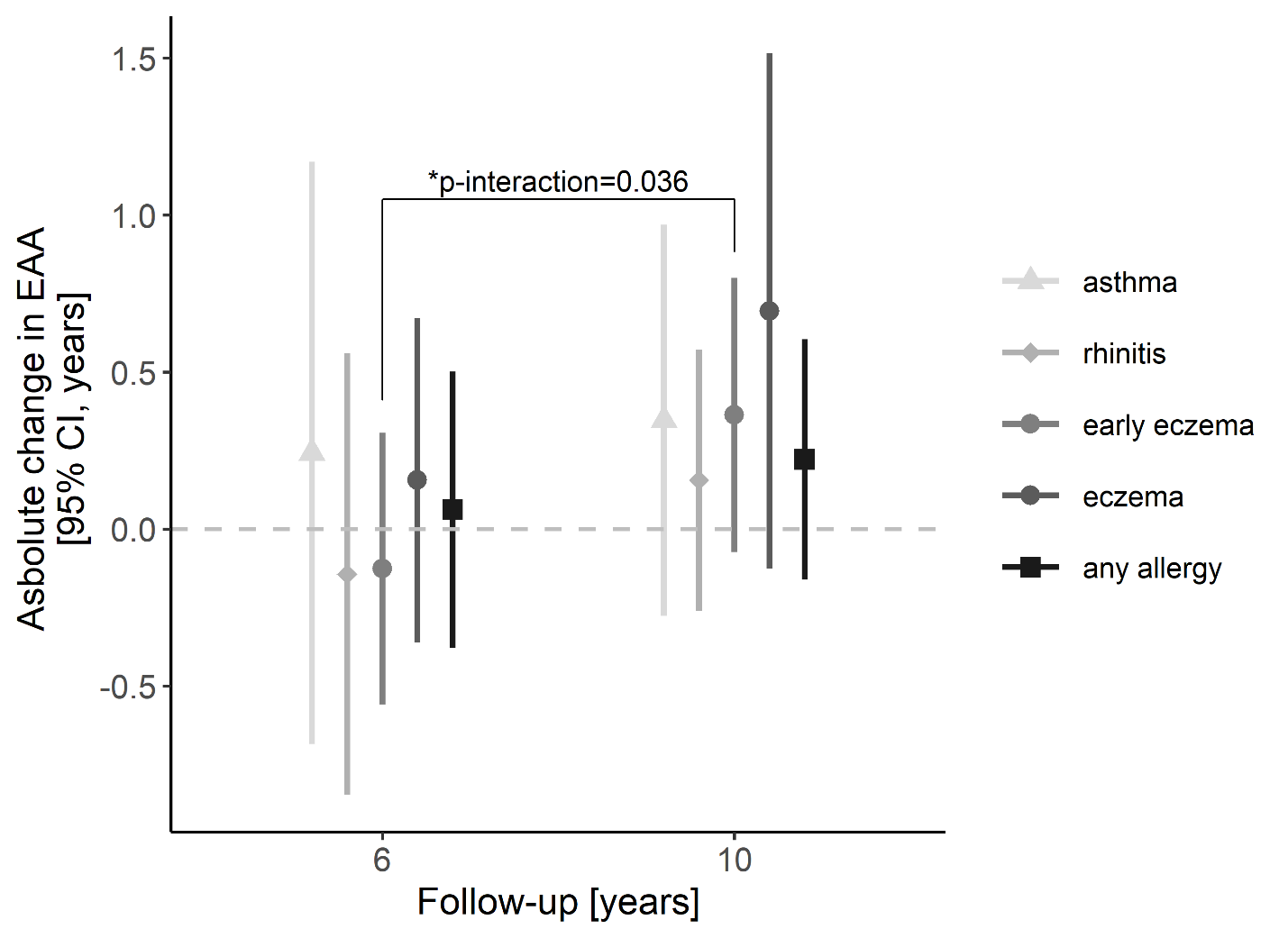


Figure S4: Linear mixed model means of EAA in LISA at the 6- and 10-year follow-up for main allergic phenotypes, including time interaction terms. *There is a significant interaction effect between allergic phenotype and follow-up time point.


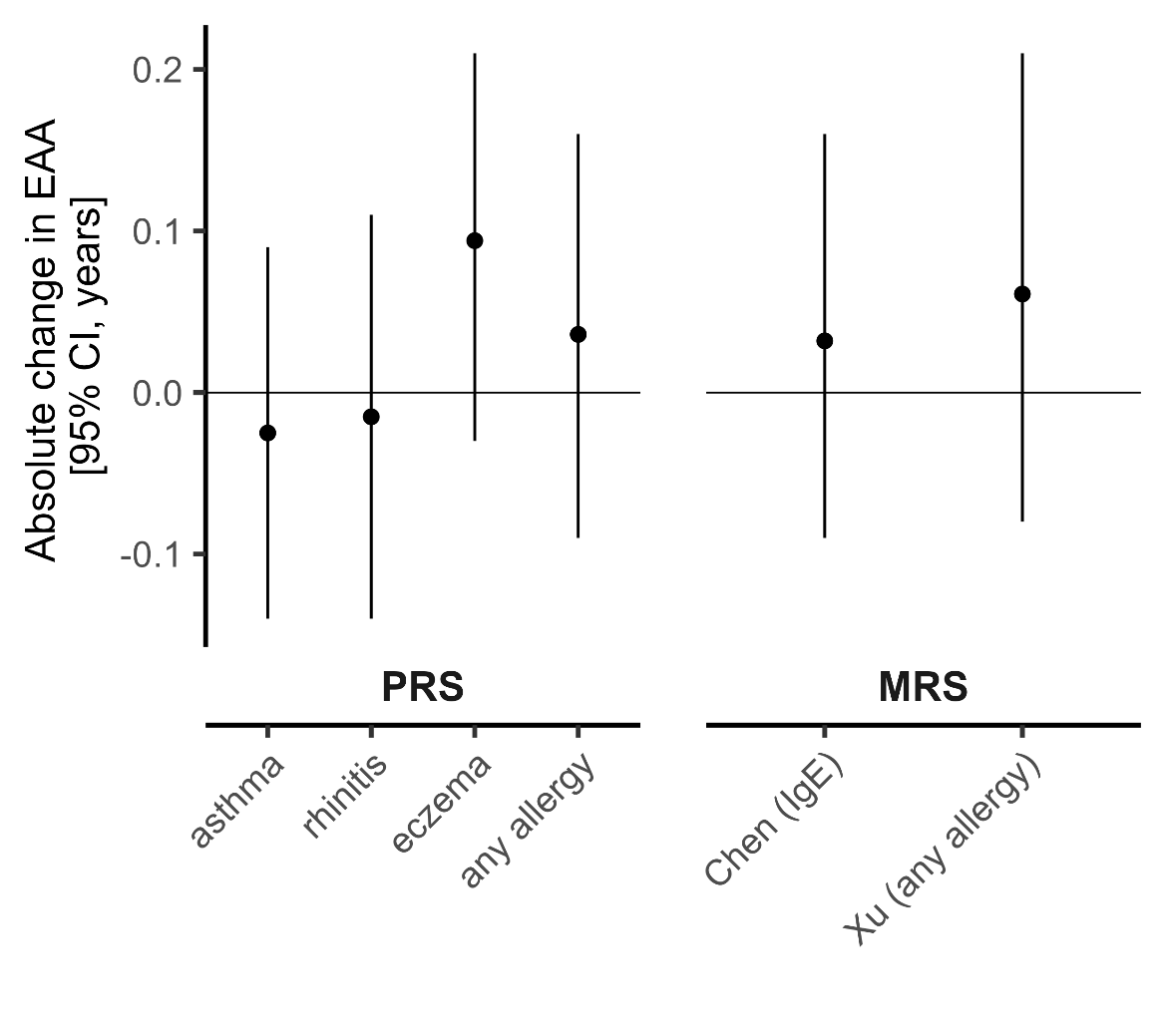


Figure S5: Associations of cell type proportion adjusted epigenetic age acceleration (EAA) in LISA with polygenic risk scores (PRS) for asthma, rhinitis, eczema, and any allergy and methylation risk scores (MRS) for IgE and any allergy.


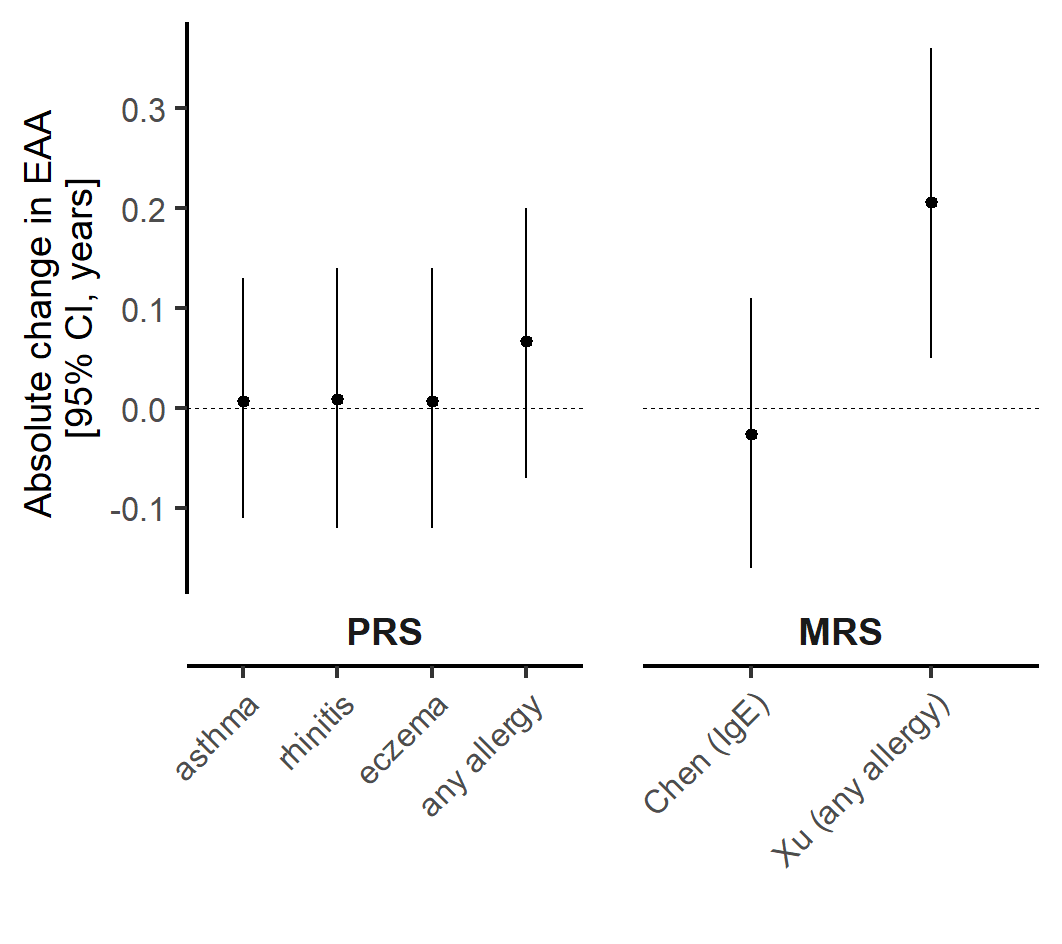


Figure S6: Associations of epigenetic age acceleration (EAA) (not adjusted for cell type proportions) in LISA with polygenic risk scores (PRS) for asthma, rhinitis, eczema, and any allergy and methylation risk scores (MRS) for IgE and any allergy.


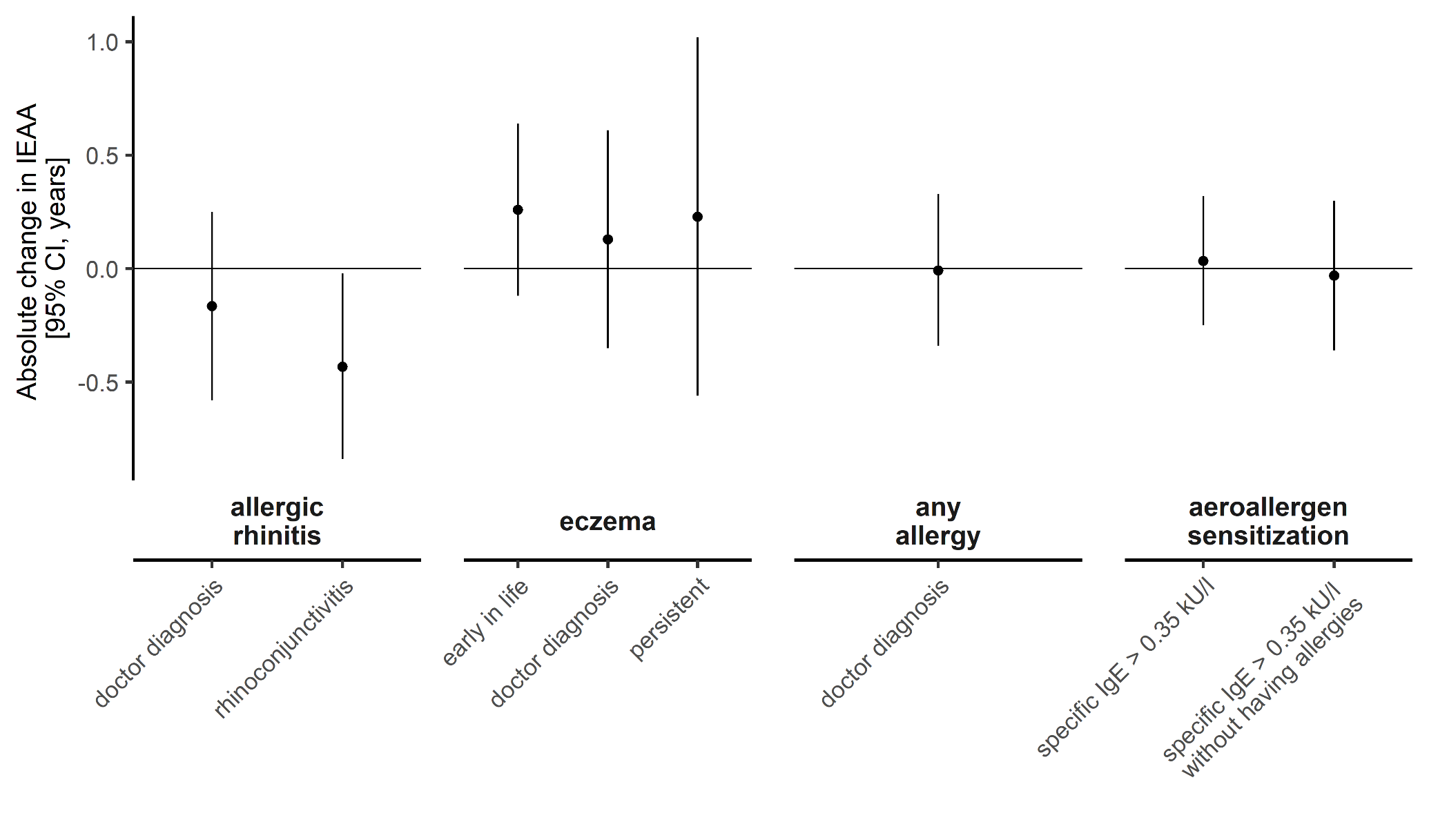


Figure S7: Intrinsic epigenetic age acceleration (IEAA) calculated by the Horvath pan-tissue clock in LISA children with allergic rhinitis, eczema, any allergy or aeroallergen sensitization.


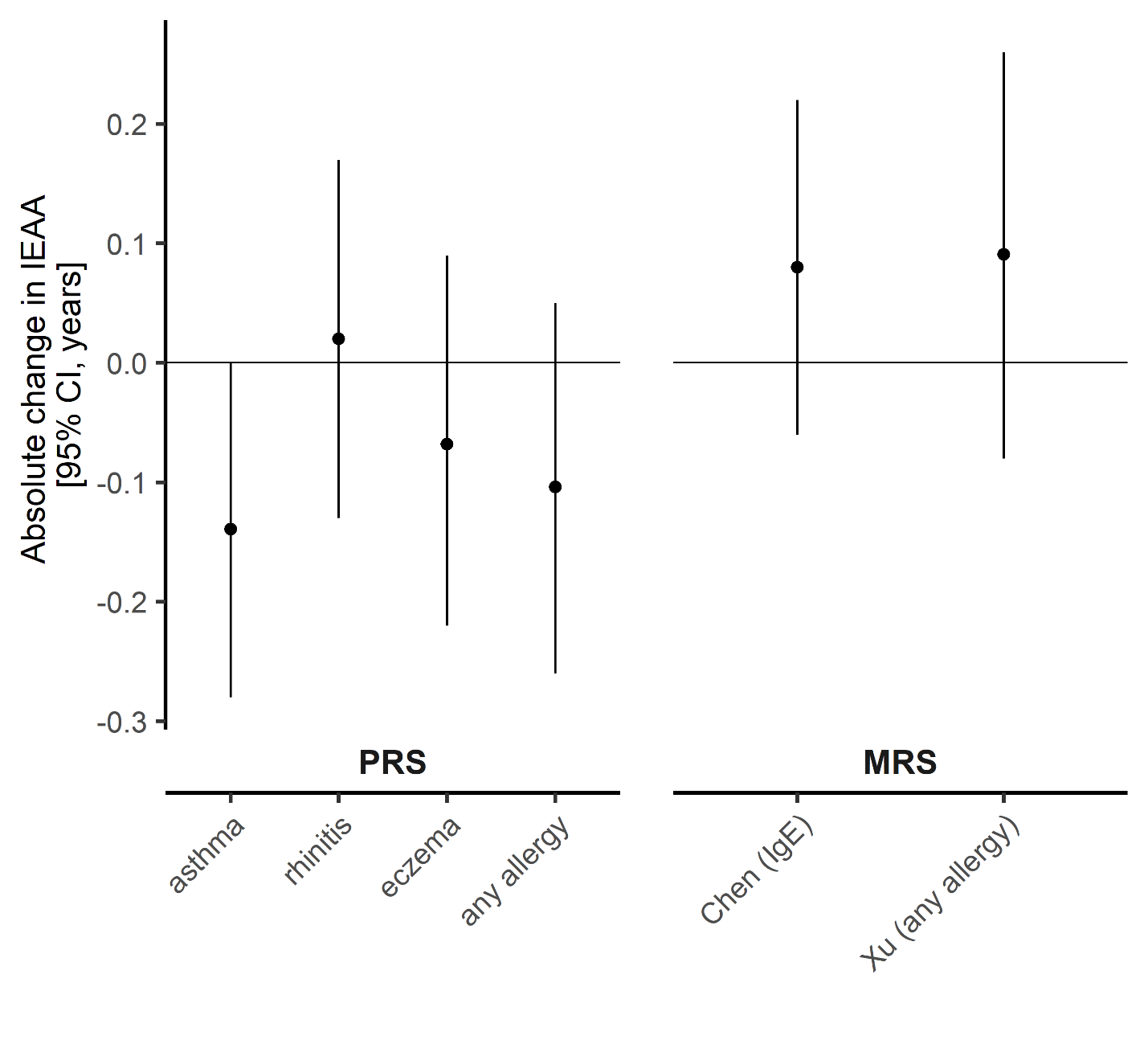


Figure S8: Associations of intrinsic epigenetic age acceleration (IEAA) in LISA calculated by the Horvath pan-tissue clock with polygenic risk scores (PRS) for asthma, rhinitis, eczema, and any allergy and methylation risk scores (MRS) for IgE and any allergy.


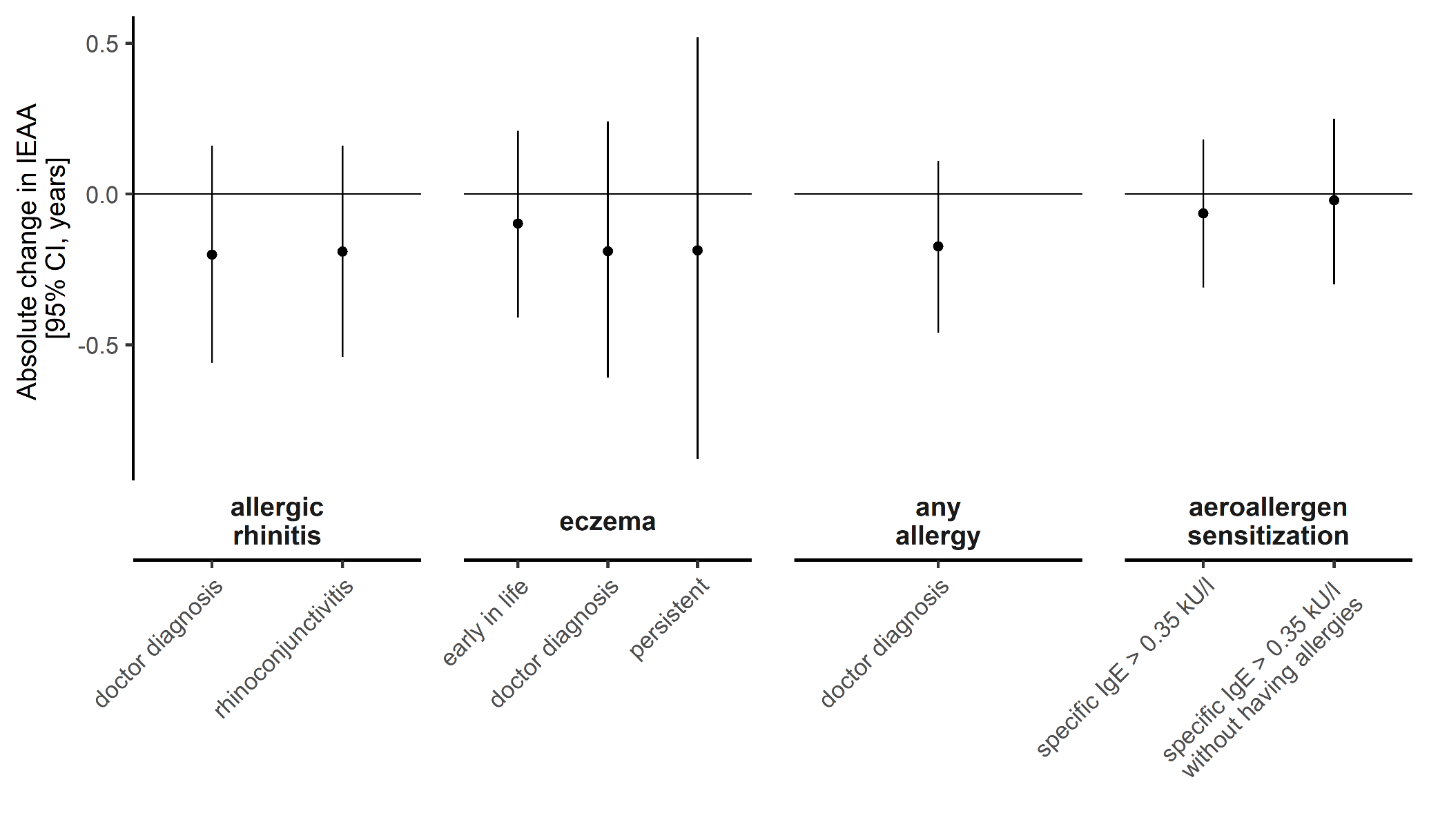


Figure S9: Intrinsic epigenetic age acceleration (IEAA) calculated by the Horvath skin&blood clock in LISA children with allergic rhinitis, eczema, any allergy or aeroallergen sensitization.


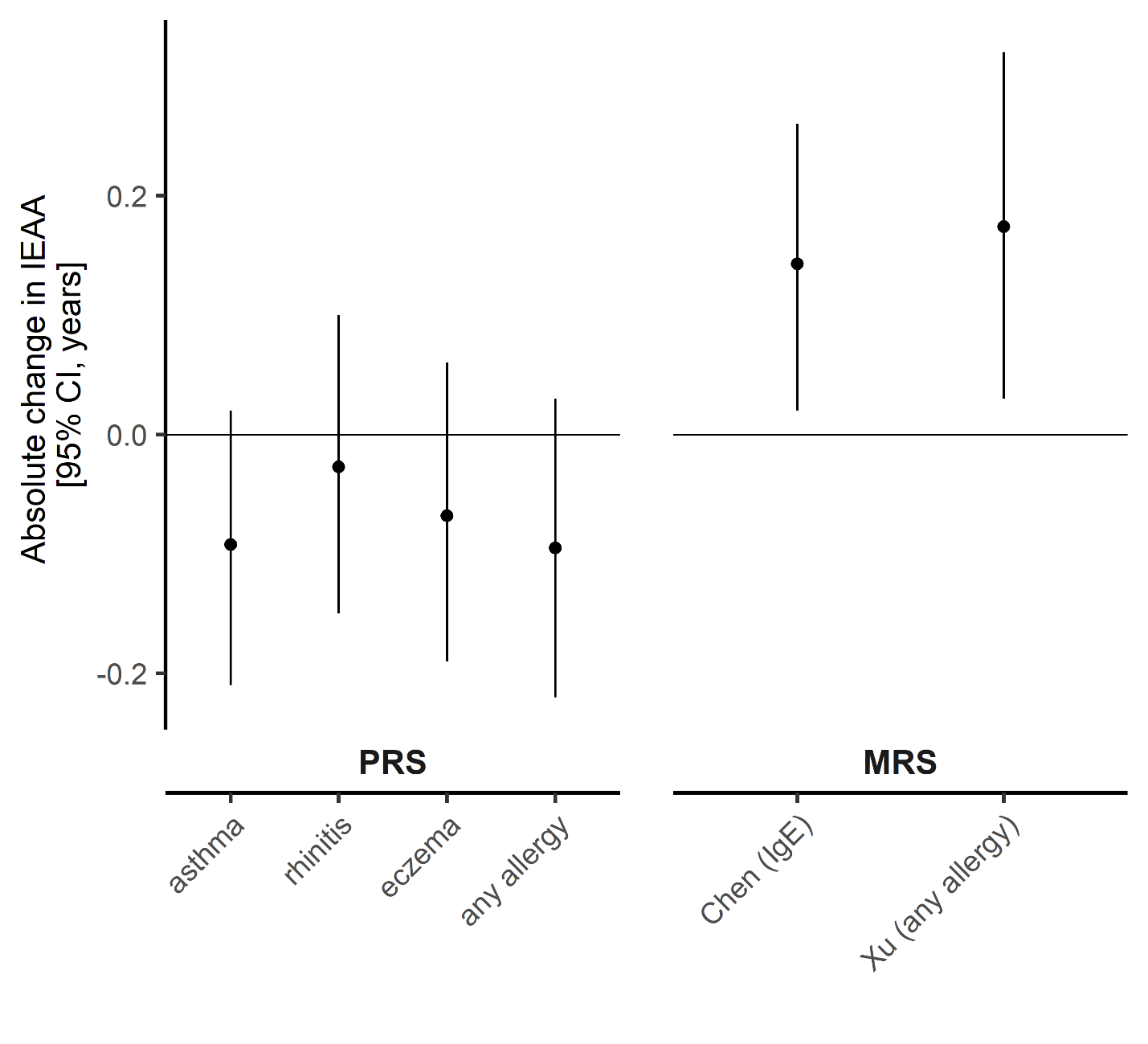


Figure S10: Associations of intrinsic epigenetic age acceleration (IEAA) in LISA calculated by the Horvath skin&blood clock with polygenic risk scores (PRS) for asthma, rhinitis, eczema, and any allergy and methylation risk scores (MRS) for IgE and any allergy.

Asthma


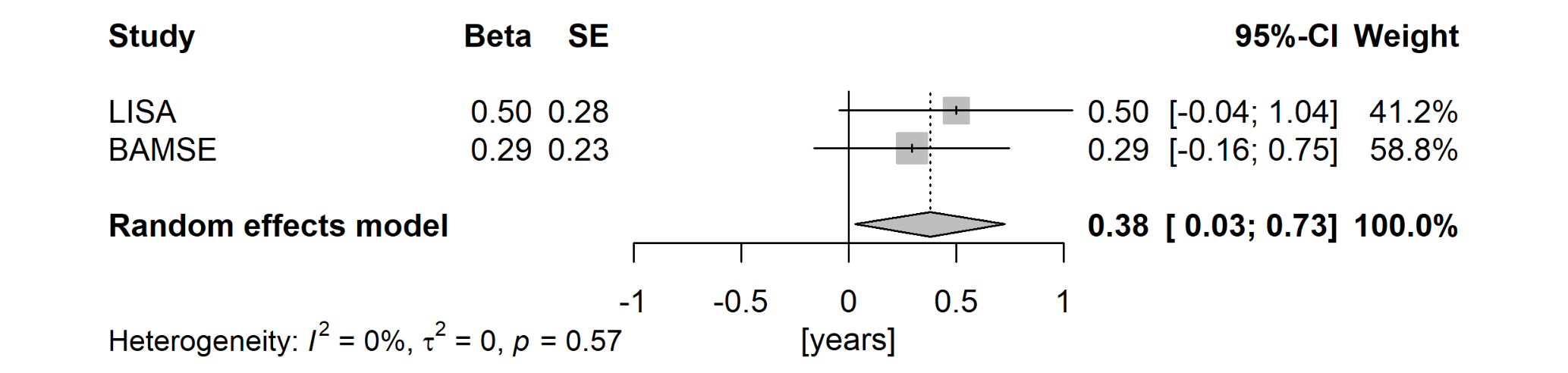


Allergic rhinitis


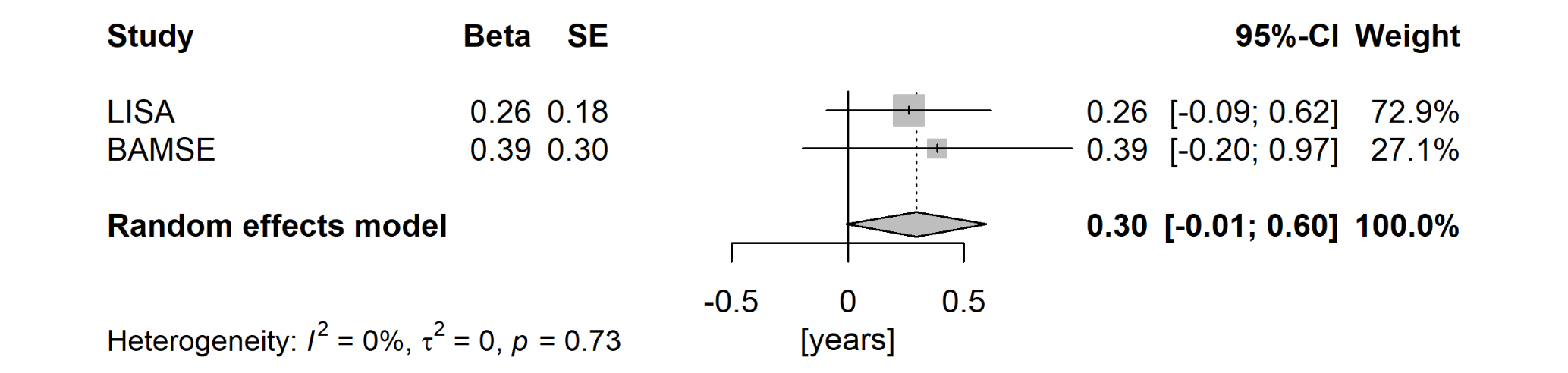


Eczema


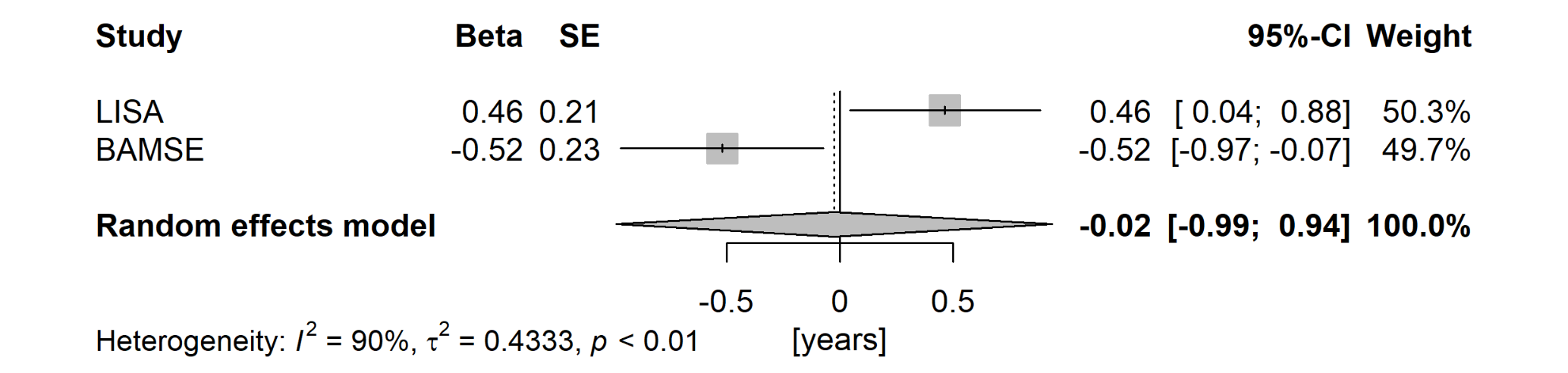


Any allergy


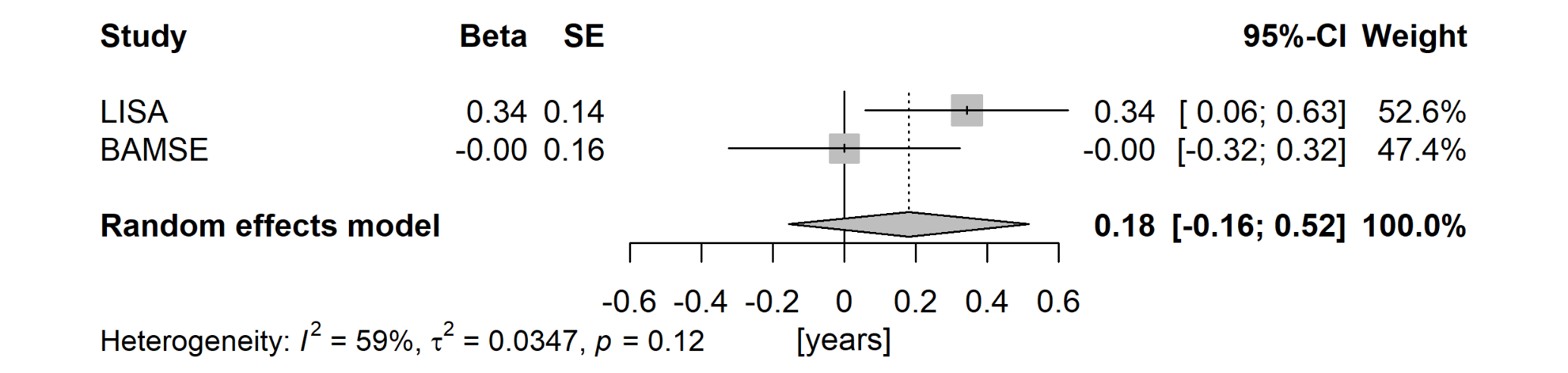


Aeroallergen sensitization


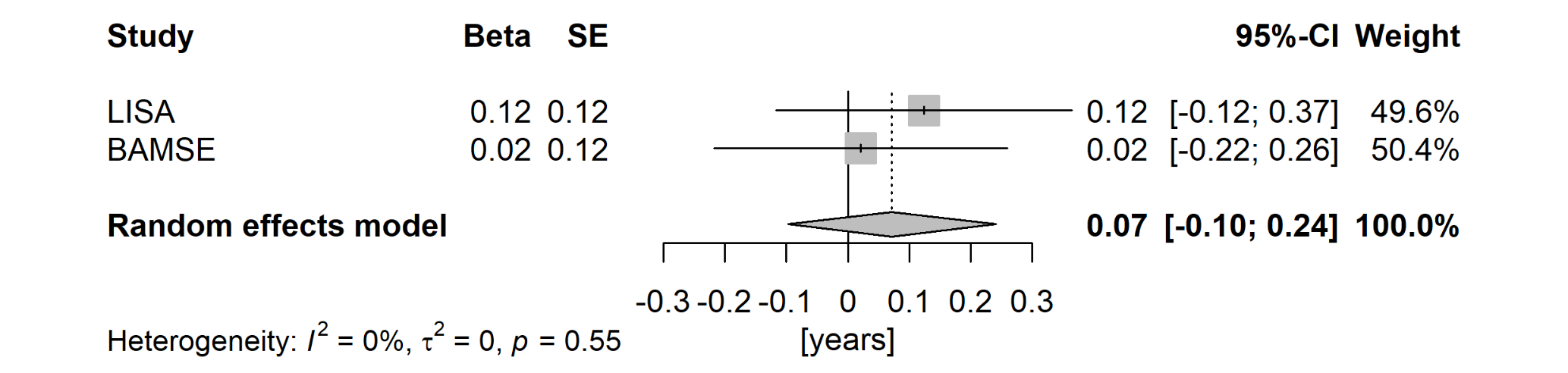


Figure S11: Random effects inverse variance meta-analysis of LISA and BAMSE results.


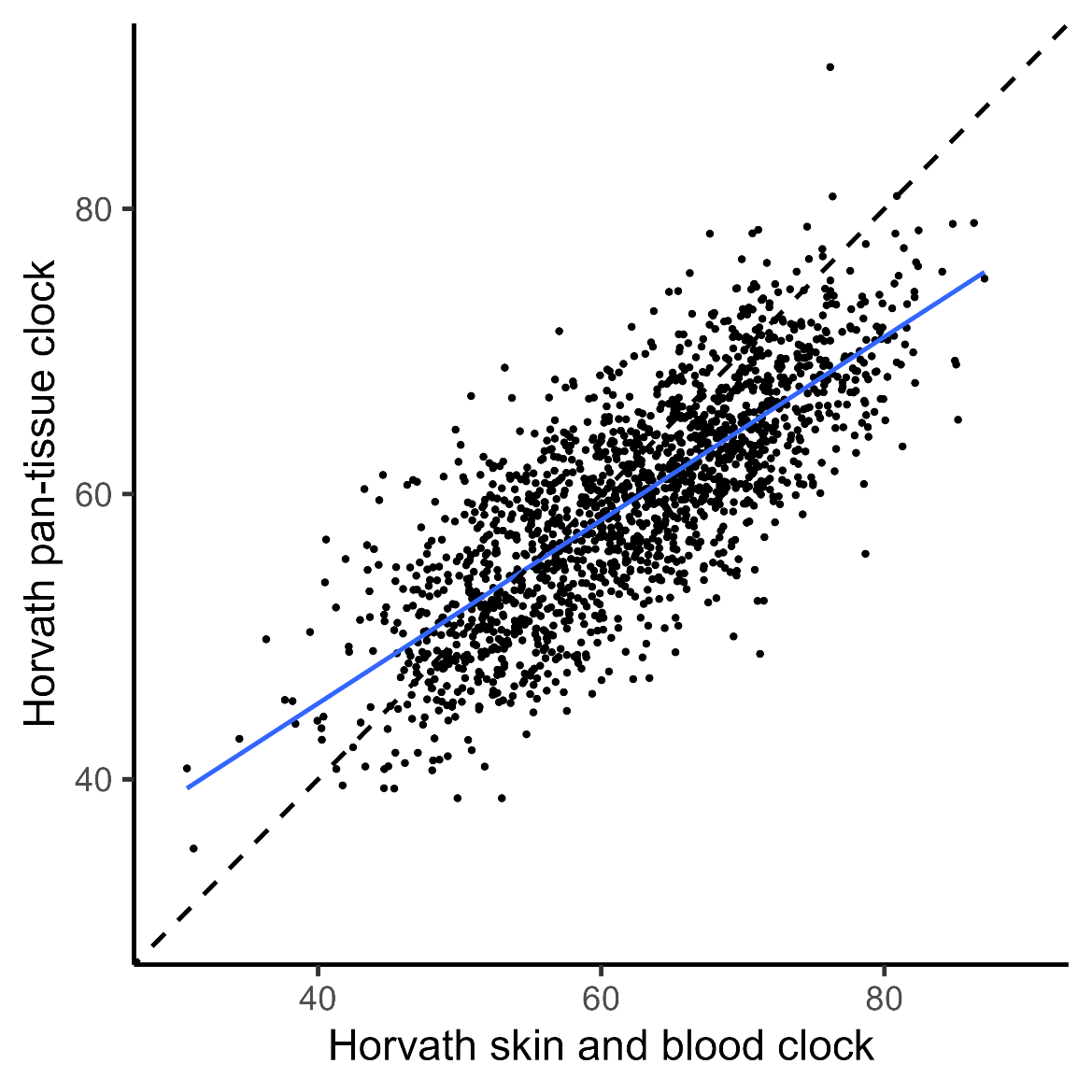


Figure S12: Correlation between the Horvath pan-tissue (y axis) and Horvath skin&blood clock (x axis) within the KORA F4 cohort. The blue line represents a linear regression line. Pearson correlation coefficient r=0.77.


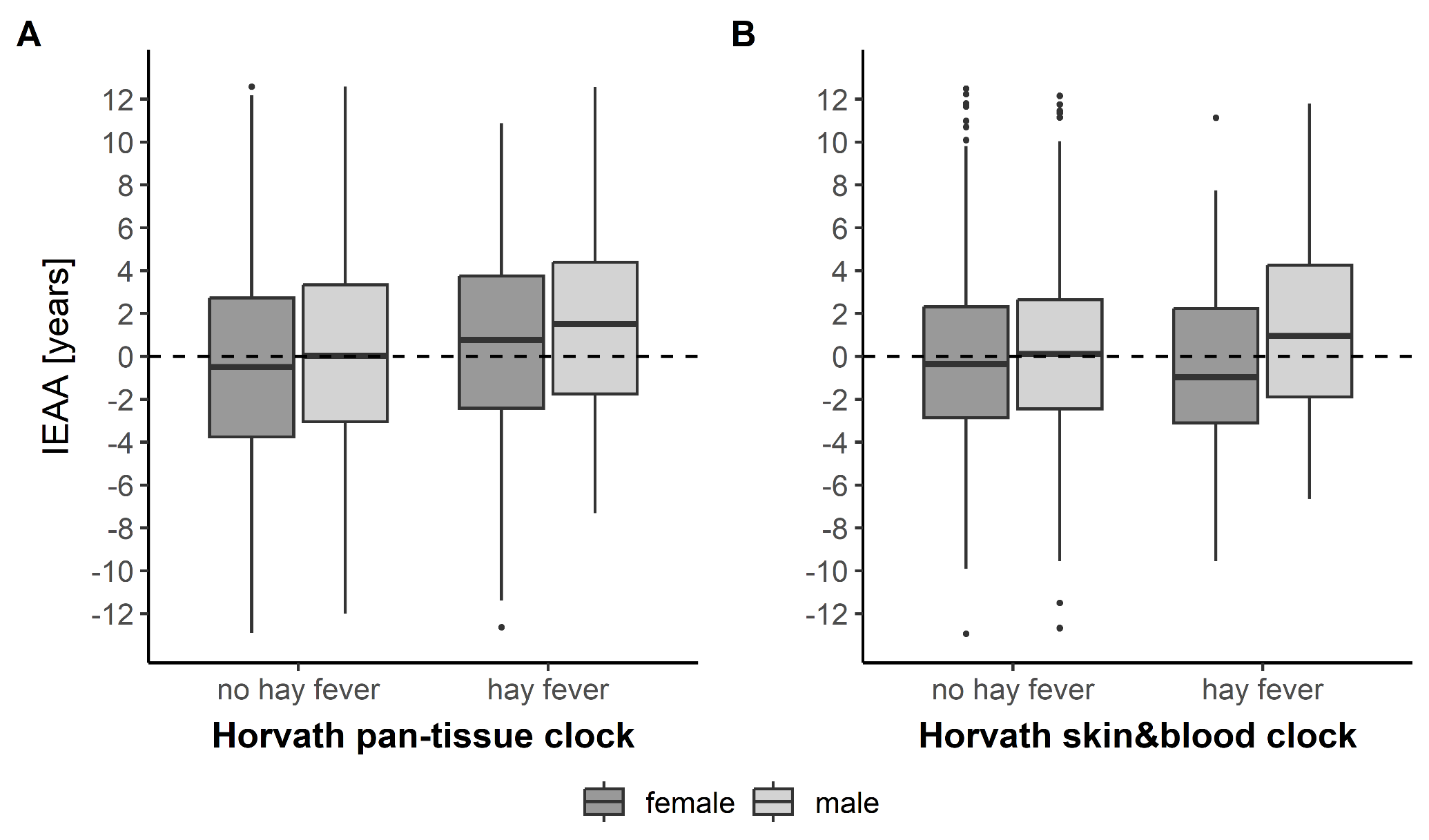


Figure S13: Descriptive comparison of intrinsic epigenetic age acceleration (IEAA) distribution in males (light grey) and females (dark grey) with or without hay fever in adults from KORA F4, (A) using the Horvath pan-tissue clock, and (B) the Horvath skin and blood clock. The interaction analysis revealed that men with hay fever were 1.72 years (95%CI [0.29;3.15]) older than women with hay fever when using the Horvath skin and blood clock (B). When using the Horvath pan-tissue clock (A), the interaction term did not reach statistical significance (0.65 years, 95%CI [-1.07;2.36]).
